# Hepatitis B Surface Antigen Directly Alters Dendritic Cell–Mediated Natural Killer Cell Responses

**DOI:** 10.1016/j.jcmgh.2026.101870

**Published:** 2026-08-29

**Authors:** Lucile Dumolard, Marie-Noelle Hilleret, Mylene Pezet, Thomas Decaens, Philippe Saas, Evelyne Jouvin-Marche, Patrice N. Marche, Caroline Aspord, Zuzana Macek Jilkova

**Affiliations:** 1 Institute for Advanced Biosciences, Inserm U 1209, CNRS UMR 5309, University Grenoble Alpes, Grenoble, France; 2 Hepato-Gastroenterology and Digestive Oncology Department, CHU Grenoble Alpes, Grenoble, France; 3 Etablissement Français du Sang Auvergne-Rhone-Alpes, R&D Laboratory, Grenoble, France

**Keywords:** Dendritic Cells, HBsAg, Hepatitis B, NK Cells

## Abstract

**Background & Aims:**

Hepatitis B, caused by the hepatitis B virus, is a significant global health concern, often leading to chronic hepatitis B in individuals unable to mount an effective immune response. Chronic hepatitis B virus substantially increases the risk of severe liver diseases, including cirrhosis and hepatocellular carcinoma. Dendritic cells and natural killer cells play crucial roles in the early immune response to hepatitis B virus, but their function is compromised in patients with chronic hepatitis B. We investigated how hepatitis B surface antigen influences the interplay between dendritic cell subpopulations and natural killer cells.

**Methods:**

Blood-derived dendritic cell subsets were pre-exposed to hepatitis B surface antigen and then cocultured with natural killer cells under various stimulation conditions. We subsequently evaluated the modulations of dendritic cells and the phenotypic and functional responses of natural killer cells. Similarly, dendritic cell subsets were exposed to serum from hepatitis B virus–infected individuals with varying levels of hepatitis B surface antigen or directly purified from patients with hepatitis B virus to assess their ability to stimulate natural killer cell responses.

**Results:**

Our findings show that hepatitis B surface antigen disrupts type 2 conventional dendritic cell–mediated natural killer cell activation through Toll-like receptor 7 and 8–dependent pathways and type 1 dendritic cell–mediated natural killer cell degranulation via Toll-like receptor 3–dependent mechanisms. Exposure of plasmacytoid dendritic cells to hepatitis B surface antigen alters natural killer cell activation, phenotype, and degranulation through Toll-like receptor 9–dependent pathways. This impairment was recapitulated following exposure to serum from hepatitis B virus–infected patients in a hepatitis B surface antigen–dependent manner. Consistently, circulating hepatitis B surface antigen in patients with chronic hepatitis B virus infection was associated with impaired plasmacytoid dendritic cell–mediated natural killer cell cytotoxicity.

**Conclusions:**

These results uncover key mechanisms by which hepatitis B surface antigen modulates plasmacytoid dendritic cell–natural killer cell interactions, shedding light on how hepatitis B virus evades innate immune responses and contributes to immune dysfunction.

## Graphical abstract

**Figure:**
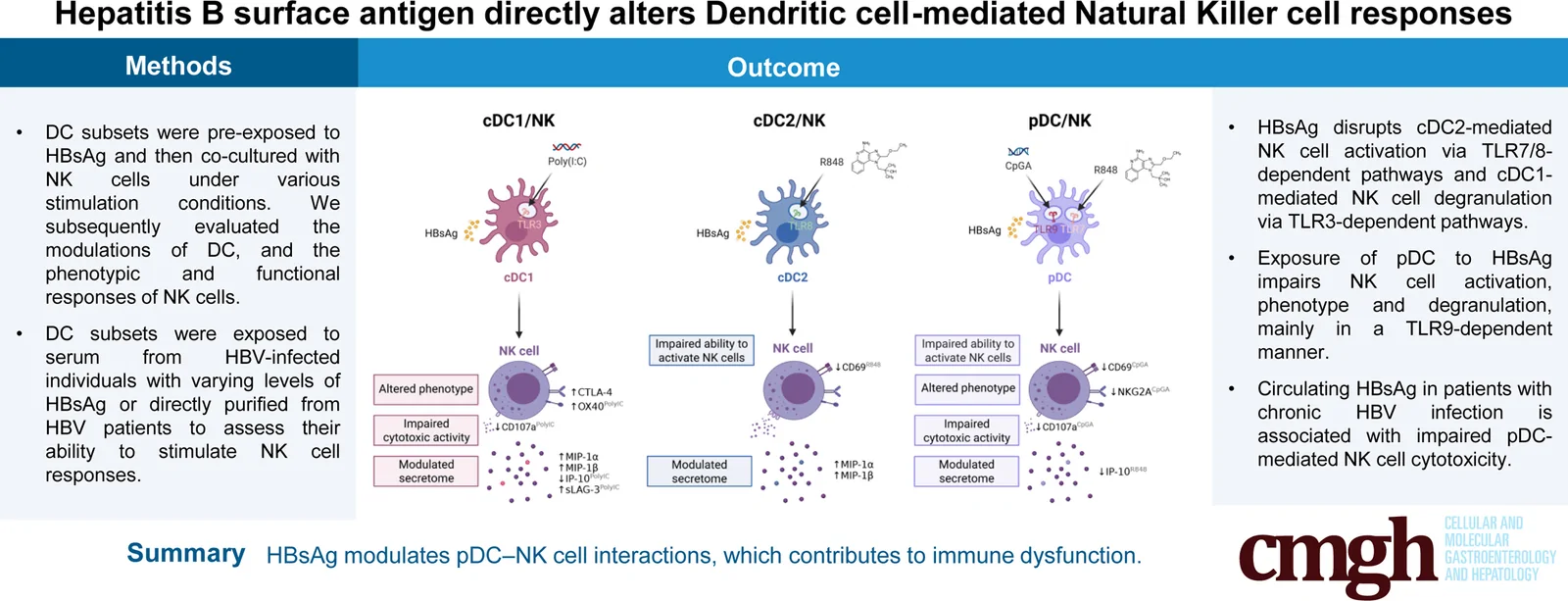

**Summary**

This study reveals how hepatitis B surface antigen disrupts dendritic cell–mediated natural killer cell responses via toll-like receptor–dependent pathways in a subset-specific manner. These findings clarify the mechanisms of hepatitis B virus immune evasion and provide insights into developing immune restoration strategies for chronic hepatitis B virus infection.

**What You Need to Know**

**Background**

Hepatitis B is associated with impaired innate immunity. We investigated how hepatitis B surface antigen modulates the ability of dendritic cells to trigger natural killer cell responses.

**Impact**

This study reveals mechanisms by which hepatitis B surface antigen disrupts dendritic cell–natural killer cell crosstalk, impairing natural killer cell activation and cytotoxic function through distinct Toll-like receptor–dependent pathways across dendritic cell subsets.

**Future Directions**

These findings highlight the critical role of distinct dendritic cell subsets in anti–hepatitis B virus immunity by orchestrating effective natural killer cell responses and support the development of targeted immunomodulatory strategies.

Despite the existence of an effective vaccine, almost 300 million people are chronically infected with hepatitis B virus (HBV) globally.^1^ Long-term chronic infections can lead to serious, life-threatening liver diseases, like cirrhosis or hepatocellular carcinoma.

The HBV pathophysiology is strongly related to the host’s immune system: approximately 90%–95% of adults infected with the virus achieve immune-mediated viral clearance. However, those who fail to generate a sufficiently robust and functional antiviral immune response progress to chronic hepatitis B (CHB).^2^ Nucleos(t)ide analogs are the most frequent therapies used to treat HBV infections. They can efficiently inhibit viral replication, but they do not provide a complete cure, and life-long nucleos(t)ide analog therapy is often required to maintain HBV suppression.^3^ Therefore, it is essential to better understand the pathophysiology of HBV chronic infections in order to design novel or complementary therapeutic strategies aimed at achieving immune control and curing patients.

Dendritic cells (DCs) are crucial components of the innate immune system, playing a role in initiating effective antiviral immunity. DCs orchestrate antiviral immune responses by sensing the HBV virus, processing viral antigens, and activating the different effector cells. There are 3 major DC subsets comprising: (1) conventional DCs (cDCs) type 1 (cDC1s) expressing BDCA3; (2) cDCs type 2 (cDC2s) expressing BDCA1; and (3) plasmacytoid DCs (pDCs) expressing the surface molecule BDCA2.^4^ DCs express distinct sets of pathogen recognition receptors, such as Toll-like receptors (TLRs) and C-type lectin receptors (CLRs), allowing them to recognize different molecular patterns expressed by pathogens and trigger the production of proinflammatory and antiviral cytokines. cDC1s secrete interferon (IFN)-λ in response to TLR3 stimulation, whereas cDC2s predominantly express TLR4 and 8 and are the main producers of interleukin (IL)12p70.^4^^,^^5^ pDCs mainly express TLR7 and 9, playing a critical role in antiviral immunity by producing type I IFNs in response to viral infections.^4^^,^^5^

Effector cells from innate and adaptive immune system (natural killer [NK] cells, T cells, and B cells) are key players to resolve the infection. NK cells are enriched in the liver and play an important role mainly in the early stages of viral infection as they have the ability to recognize and directly kill virus-infected cells through cytotoxic granules and to release antiviral cytokines.^6^ NK cells can be categorized into 2 distinct subsets: CD56^dim^CD16^+^ cells, which are highly cytotoxic and primarily circulate in peripheral blood, and CD56^high^CD16^-^ cells, which primarily reside in the liver and are specialized for cytokine production.

TLRs expressed by DCs play a central role in initiating and shaping DC–NK cell crosstalk. Upon recognition of pathogen-associated signals, DCs produce key cytokines such as IL12, IL15, and IL18, which are critical for NK cell activation, proliferation, and effector function. Several studies further show that TLR stimulation enhances DC–NK interactions and promotes NK cell activation,^7^^,^^8^ highlighting the importance of TLR-driven signaling pathways in coordinating innate immune responses.

In HBV infection, the interplay between DCs and NK cells is critical for the regulation of the immune response. However, both DCs and NK cells are known to be dysregulated in chronic HBV, which can lead to ineffective viral clearance and persistent infection.^2^

In patients with chronic HBV, both circulating and intrahepatic DCs exhibit significant phenotypic and functional alterations.^9^^,^^10^ These include diminished expression of costimulatory molecules, altered TLR and CLR profiles, and consequently, impaired cytokine production.^11^, ^12^, ^13^, ^14^, ^15^ We recently demonstrated that HBV and its viral protein released by HBV-infected hepatocytes—hepatitis B surface antigen (HBsAg)—directly trigger functional defects in cDC1s, cDC2s, and pDCs that might contribute to the failure to properly elicit antiviral immune responses.^16^

NK cells also exhibit functional impairments and features of exhaustion during HBV infection, including decreased cytokine production, which contributes to viral persistence.^17^, ^18^, ^19^, ^20^ NK cell cytotoxicity in chronic HBV infection was reported to be either impaired^21^^,^^22^ or conserved,^17^, ^18^, ^19^ depending on the HBV phase and the approach used to investigate effector functions.^20^, ^21^, ^22^, ^23^ The functional impairment of NK cells in chronic HBV infection is further reflected in the elevated expression of immune checkpoint molecules (ICMs), like programmed cell death 1 (PD-1), inducible T cell costimulator (ICOS), and 4-1BB on NK cells from patients with chronic HBV,^17^^,^^24^^,^^25^ similar to what is observed in T cells.

Several studies indicated that HBsAg levels in patients with HBV may impact NK phenotype and function. The elevated levels of serum HBsAg in patients with chronic HBV infection were associated with a less activated phenotype^23^ and the diminished cytotoxicity of peripheral NK cells,^26^ as well as decreased activation of intrahepatic NK cells.^19^

DCs play a critical role in stimulating NK cells through a variety of mechanisms that enhance NK cell activation and function. DCs secrete cytokines and chemokines (like CXCL10) that are crucial for NK cell activation. DCs can also interact with NK cells via cell–cell contact involving different molecules, including CD40 and CD86, or ICM pathways like ICOS and ICOS ligand, glucocorticoid-induced tumor necrosis factor receptor–related protein (GITR)/GITR ligand or OX-40/OX-40 ligand.^22^^,^^27^, ^28^, ^29^, ^30^ Therefore, NK cells’ dysfunctions observed in patients with chronic HBV may be partially the result of an initial altered triggering by DCs.

A recent study highlighted that HBV disrupts the crosstalk between DCs and NK cells, impairing their functional interaction.^31^ Similarly, an earlier work demonstrated that pDCs derived from patients with chronic HBV failed to induce NK cell activation, cytokine production, and cytolytic activity,^22^ emphasizing the critical role of reciprocal interactions between innate immune cells.

However, the specific impact of HBsAg on the capacity of DC subsets to trigger NK cells remains unclear and requires further exploration. Therefore, we investigated the impact of HBsAg on various DC subsets and the consequences on subsequent NK cell activity and functionality, as well as the significance of this altered interaction in the context of chronic HBV infection.

## Results

### Hepatitis B Surface Antigen Modulates Dendritic Cell Subsets’ Features

First, we investigated how HBsAg affects the DC expression of molecules involved in the crosstalk with NK cells. PanDCs (all subsets of DCs) were fluorescence-activated cell sorting (FACS)-sorted and exposed to HBsAg for 20 hours, followed by RNA sequencing analysis (Figure 1*A*). The heatmap of differentially expressed genes revealed a clear distinction between DCs exposed or not to HBsAg (Figure 1*B*). The volcano plot highlights the most significantly upregulated and downregulated genes after HBsAg exposure (Figure 1*C*). Notably, selected genes critical for crosstalk with NK cells exhibited significant changes in their expression, with both upregulation and downregulation observed (Figure 1*D*). Specifically, exposure to HBsAg resulted in a marked decrease in the gene expression of the chemokines C-C chemokine receptor type 2 (*CCR2*) and C-C chemokine ligand type 2 (*CCL2*) (Figure 1*D*), whereas the expression of *CCL19, CXCL2, CXCL1, CXCL8*, and *CCL22* was increased (Figure 1*D*; Supplementary Table 1). These findings suggest that HBsAg indirectly modulates chemotactic signals, potentially altering the recruitment of immune cells, including NK cells, to the site of infection. Additionally, we observed a significant reduction in the major inflammatory cytokine gene *IL18*, which is crucial for NK activation, whereas *IL12* and *IL15* were slightly upregulated (Figure 1*D*; Supplementary Table 1). ICMs also showed changes, with a reduction in the TIM-3/Gal9 pathway, and an increase in *PD-L1* and *4-1BBL* gene expression (Figure 1*D*; Supplementary Table 1). Overall, these modulations of immune signaling molecules by HBsAg could influence the DC–NK cell crosstalk and thus the antiviral response.

**Figure 1:**
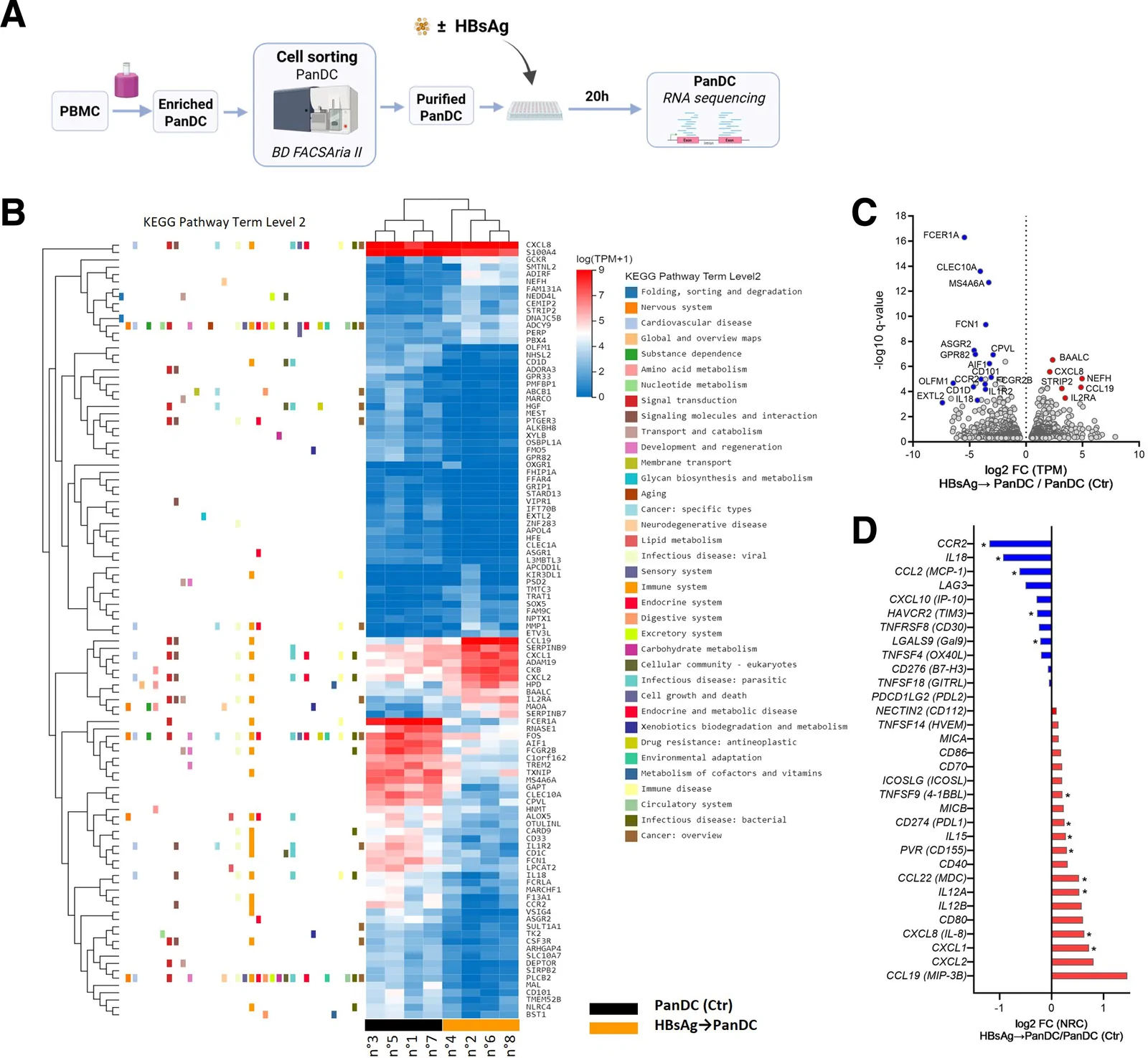
**HBsAg impacts molecules involved in DC–NK crosstalk.** FACS-sorted PanDCs from HDs were exposed or not to HBsAg and submitted to RNA sequencing. (*A*) Schematic representation of the experimental layout used to investigate the impact of HBsAg on cell-sorted PanDCs by RNA sequencing. Created in BioRender. (*B*) The heatmap of differentially expressed genes presented as transcript per million clustering (TPM) for nonstimulated PanDC samples (Ctr; *black*) and PanDCs pre-exposed to HBsAg (*orange*). (*C*) The volcano plot highlighting the most significantly upregulated (*red*) and downregulated (*blue*) genes after HBsAg exposure. (*D*) Fold change (FC) of normalized read count (NRC) of selected genes critical for crosstalk with NK cells, with upregulated (*red*) and downregulated (*blue*) genes. ∗*P* value < .05. N = 4 independent experiments (4 different HDs used for isolation of cells). KEGG, Kyoto Encyclopedia of Genes and Genomes.

Next, we explored how HBsAg affects DC subset activation. PanDCs were exposed to HBsAg and stimulated (or not) with Toll-like receptor ligand (TLR-L), monitoring the expression of costimulatory molecules CD40, CD80, and CD86 (Figure 2*A*). Heatmaps of fold changes in these markers compared with controls (no HBsAg, no stimulation) revealed HBsAg’s direct effects and its impact on TLR-dependent responses. Without TLR stimulation, HBsAg strongly activated all DC subsets, increasing CD40, CD80, and/or CD86 expression (Figure 2*B*; Supplementary Table 2). Upon TLR3 (polyinosine-polycytidylic acid [Poly(I:C)]) stimulation, HBsAg reduced the upregulation of CD80 levels in cDC2s and CD40 and CD80 levels in pDCs. Upon TLR7 and 8 (R848) stimulation, HBsAg reduced CD40 and CD80 levels in cDC2s and CD80 levels in pDCs. On the other hand, the expression levels of CD40 and/or CD80 were slightly enhanced by HBsAg in all DCs stimulated with TLR9 (class A CpG oligodeoxynucleotide [CpGA]) (Figure 2*B*). These findings demonstrate that HBsAg activates DC subsets and modulates their TLR-stimulated responses.

**Figure 2:**
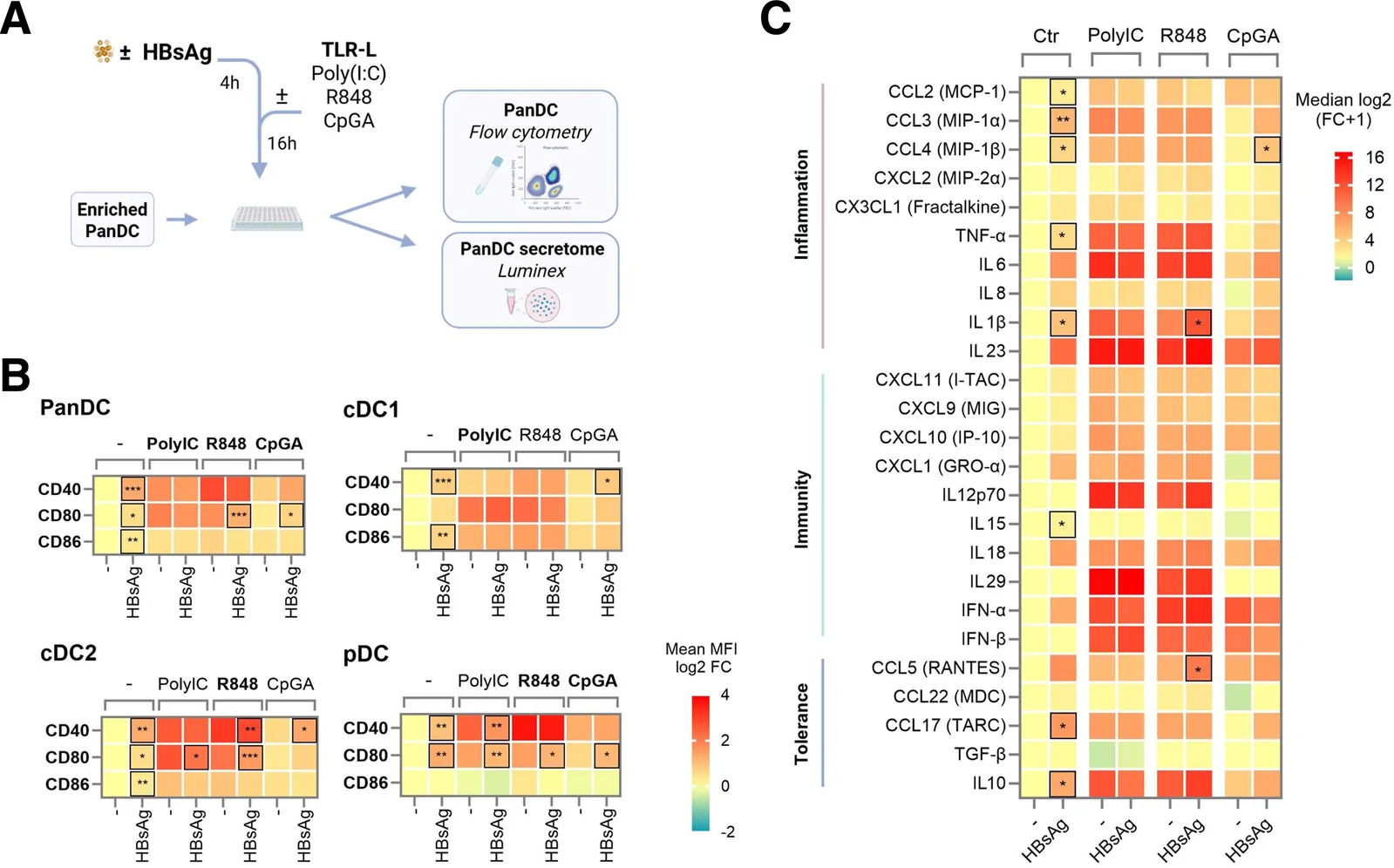
**HBsAg modulates activation and secretome of DCs.** PanDCs purified from HDs were exposed or not to HBsAg, followed by stimulation or no stimulation with TLR-L. The activation status of DC subsets was assessed using flow cytometry, and secreted molecules were analyzed using Luminex technology. (*A*) Schematic representation of the experimental layout used to investigate the impact of HBsAg on DC activation and secretome, created in BioRender. PanDCs purified from HDs were cultured in the presence or not of HBsAg for 4 hours, and TLR3-, 7-, 8-, and 9-L were then added for 16 hours. The subsequent modulation of activation markers (CD40, CD80, CD86) on DC subsets was measured by flow cytometry. (*B*) Heatmaps based on the fold change (FC) of the averaged mean fluorescence intensity (MFI) of surface markers expressed by PanDCs, cDC1s, cDC2s, and pDCs without (−) or with HBsAg, stimulated or not by TLR-L. Reference value, no stimulation without HBsAg, was settled as blank. *Asterisks* indicate significant differences within subgroups of HBsAg–pre-exposed compared with the condition without HBsAg. ∗*P* < .05, ∗∗*P* < .01, ∗∗*P* < .001. N = 10 independent experiments (10 different HDs used for isolation of cells). (*C*) Heatmaps based on the FC of the median concentration of cytokines and chemokines secreted by PanDCs without (−) or with HBsAg exposure, stimulated or not by TLR-L. Reference value, no stimulation without HBsAg, was settled as blank. *Asterisks* indicate significant differences within subgroups of HBsAg–pre-exposed DCs compared with the condition without HBsAg. The *P* value was calculated between HBsAg and control condition for each TLR-L condition. ∗*P* < .05, ∗∗*P* < .01. N = 10–15 independent experiments.

We then investigated the impact of HBsAg on the cytokine and chemokine secretion profiles of DCs. In the culture supernatants of DCs, we analyzed 25 distinct analytes that play a role in DC–NK crosstalk and the orientation of immune responses. A heatmap, generated using the median fold change of these analytes relative to the control condition (no HBsAg, no stimulation), highlights both the direct modulatory effects of HBsAg and alterations in the DC secretome upon TLR activation (Figure 2*C*; Supplementary Table 3). HBsAg itself induced the production of proinflammatory and immunity-related cytokines and chemokines by DCs, with significant increase of CCL2, CCL3, CCL4, tumor necrosis factor-α (TNF-α), IL1β, and IL15 (Figure 2*C*). Additionally, HBsAg induced the robust secretion of regulatory chemokines such as TARC, and IL10 by DCs, even in the absence of TLR stimulation (Figure 2*C*). HBsAg strongly enhanced the production of the regulatory chemokine CCL5 and increased the secretion of the inflammatory cytokine IL1β upon R848 stimulation, as well as CCL4 in response to CpGA stimulation (Figure 2*C*). Collectively, these findings indicate that HBsAg reshapes the cytokine and chemokine secretion profiles of DCs, which can alter their subsequent interactions with NK cells.

### Hepatitis B Surface Antigen Modulates the Ability of Dendritic Cell Subsets to Activate Natural Killer Cells

Next, we explored whether HBsAg preincubation modulates the subsequent crosstalk of DCs with NK cells. DCs pre-exposed to HBsAg were cocultured with NK cells, and the phenotype, activation status, and degranulation activity of the NK cells were assessed using flow cytometry (Figure 3*A*). Additionally, cytokine and chemokine secretion profiles from the DC and NK cell cocultures were evaluated using Luminex. For comparison, NK cells were cultured alone or stimulated with IL12 and IL18, serving as negative and positive controls, respectively.

**Figure 3:**
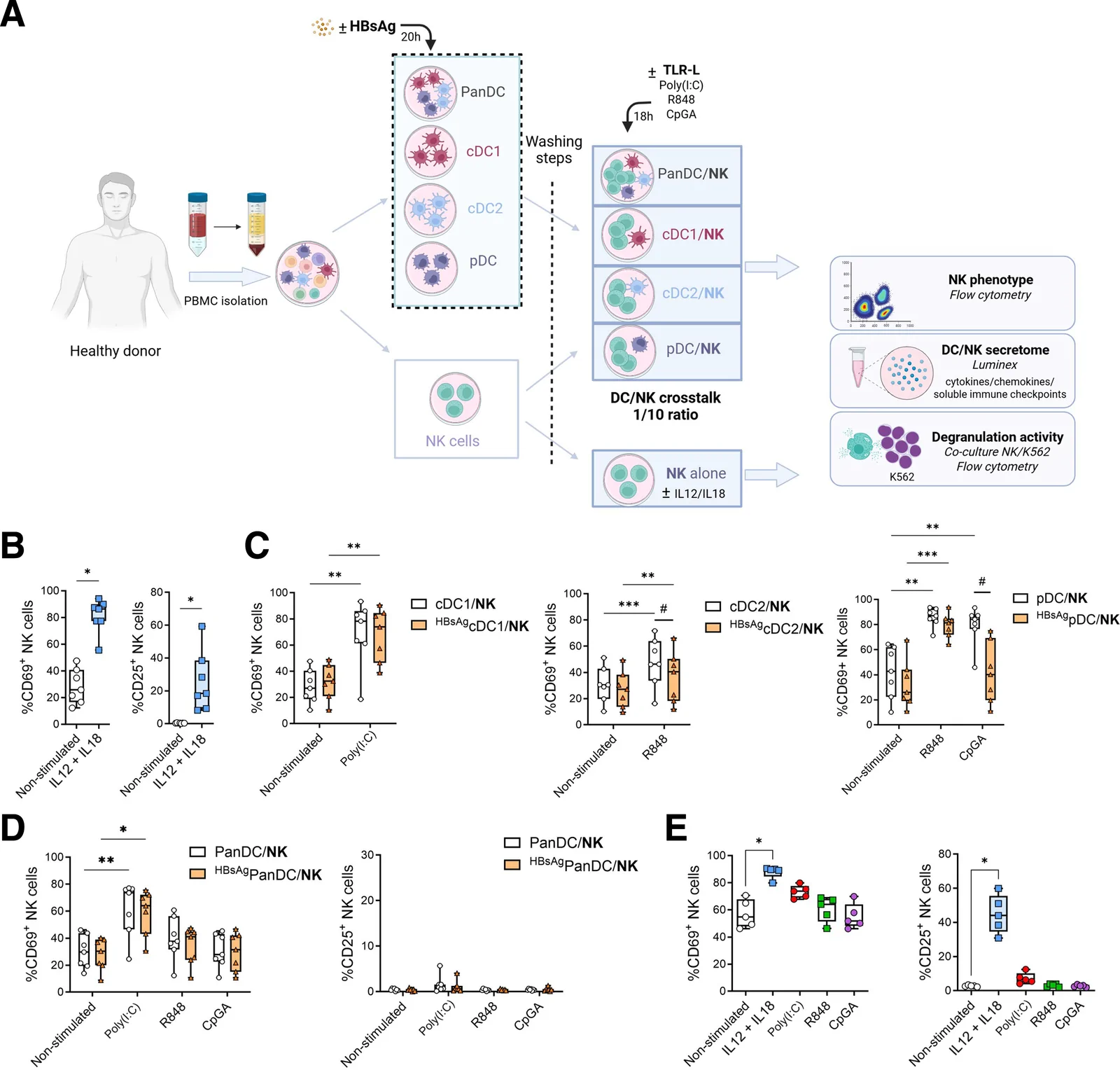
**HBsAg impairs the DC/NK cell crosstalk: the activation status of NK cells.** PanDCs purified from HDs were exposed or not to HBsAg and further cocultured with NK purified from HDs. NK cells’ phenotype and cytotoxicity were further analyzed by flow cytometry and the secretome by Luminex. (*A*) Schematic representation of the experimental layout used to investigate the impact of HBsAg on capacity of various DC subsets to modulate the NK cells’ activity, phenotype, and secretome. Created in BioRender. DCs pre-exposed to HBsAg were cocultured with NK cells in a ratio of 1:10, and the phenotype, activation status, and degranulation activity of the NK cells were assessed using flow cytometry. The secretome was analyzed by Luminex. (*B*) The frequency of CD69^+^ and CD25^+^ NK cells cultured alone, either nonstimulated or stimulated with IL12 and IL18. (C) The frequency of CD69^+^ NK cells cocultured with cDC1s, cDC2s, and pDCs, in absence (nonstimulated) or presence of TLR-L. (*D*) The frequency of CD69^+^ and CD25^+^ NK cells cocultured with PanDCs. Data represents 6 independent experiments with 6 different HDs used for cell isolation. ∗*P* < .05, ∗∗*P* < .01, ∗∗∗*P* < .001 were calculated between nonstimulated and stimulated condition, ^#^*P* < .05 was calculated between HBsAg and control condition for each TLR-L condition. (*E*) The frequency of CD69^+^ NK cells and CD25^+^ NK cells cultured alone and nonstimulated or stimulated with different TLR agonists, with N = 5 independent experiments with 5 different HDs used for NK cell isolation. ∗*P* < .05.

We first evaluated the impact of HBsAg on the ability of DCs to activate NK cells. As expected, under control conditions, IL12 and IL18 stimulation resulted in a significantly higher frequency of activated NK cells (CD69^+^ NK cells and CD25^+^ NK cells) compared with unstimulated NK cells (Figure 3*B*).

In the absence of TLR stimulation, no difference in NK cell activation was observed between coculture with DC subsets preincubated with HBsAg and those not preincubated (Figure 3*C*). TLR stimulation of DCs led to a marked increase in the frequency of CD69^+^ NK cells across all DC subsets (Figure 3*C*). However, preincubation of DCs with HBsAg substantially altered this response. Specifically, pre-exposure of cDC2s to HBsAg slightly reduced their capacity to induce NK cell activation upon TLR7 and 8 (R848) stimulation. Moreover, pre-exposure of pDCs to HBsAg strongly impaired their ability to activate NK cell upon CpGA stimulation. These findings highlight the modulatory effects of HBsAg on DC–NK cell interactions, particularly by attenuating the activation potential of cDC2 and pDC subsets in the condition of TLR7 and 8 and TLR9 stimulation, respectively. Notably, this attenuated NK cell activation was observed only when DC subsets were separately cocultured with NK cells (Figure 3*D*), suggesting a potential compensatory role of cDC1s, which remained unaffected. Furthermore, no induction of CD25^+^ expression on NK cells by DCs was detected (Figure 3*D*).

The TLR agonists used in this study target receptors predominantly expressed on DCs and not present on NK cells. To exclude the possibility of direct NK cell activation by these agonists, NK cells were cultured alone with each TLR agonist. None of the agonists enhanced NK cell activation (Figure 3*E*), confirming that the activation of NK cells observed in coculture experiments was mediated by DCs.

Altogether, these data underscore the modulatory effects of HBsAg on DC–NK cell interactions, specifically highlighting its inhibitory impact on cDC2 and pDC subsets during TLR8 and TLR9 stimulation. Although cDC1 function remained intact, the impaired ability of cDC2s and pDCs to activate NK cells suggests a potential mechanism by which HBV could evade innate immune responses.

### Hepatitis B Surface Antigen Exposure of Dendritic Cells Modulates the Phenotype of Natural Killer Cells

To better understand the impact of HBsAg on the DC-triggered NK cell phenotype, we analyzed the NK cells following coculture with different DC subsets, either pre-exposed or not to HBsAg. Our investigation centered on costimulatory molecules, key receptors that regulate NK cell activity, ICMs, inhibitory and activating NK cell receptors (NKG2 family) and natural cytotoxicity receptors. The control conditions of NK stimulation by IL12 and IL18 resulted in a significantly higher frequency of NK cells expressing PD-1, TIM-3, OX-40, 4-1BB, ICOS, and NKG2A compared with unstimulated NK cells (Figure 4*A*).

**Figure 4:**
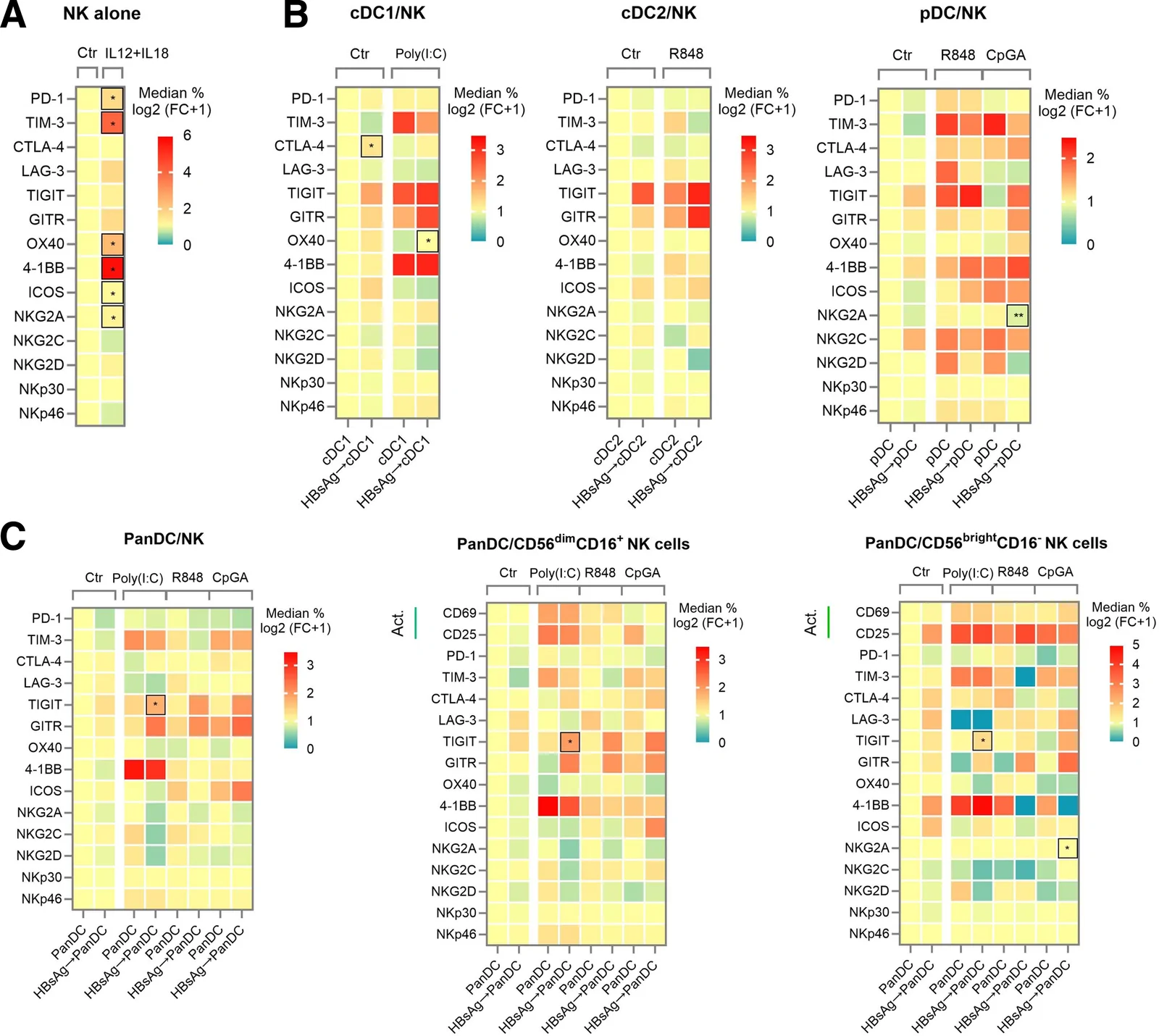
**HBsAg impairs the DC–NK cell crosstalk: the phenotype of NK cells.** DCs were exposed or not to HBsAg and further cocultured with NK cells purified from HDs. NK cells’ phenotype was analyzed by flow cytometry. (*A*) Heatmap of median frequencies of positive NK cell cultured alone, either nonstimulated or stimulated with IL12 and IL18, analyzed by flow cytometry. (*B*) Heatmap of median frequencies of positive NK cells cocultured with HBsAg pre-exposed cDC1s, cDC2s, or pDCs. (*C*) Heatmap of median frequencies of NK cells, CD56^dim^CD16^+^ NK cells, or CD56^bright^CD16^−^ NK cells cocultured with HBsAg pre-exposed PanDCs or not. Data represents 7 independent experiments (7 different HDs used for isolation of cells). The *P* value was calculated between HBsAg and control condition for each TLR-L condition. ∗*P* < .05, ∗∗*P* < .01. FC, fold change.

We next assessed how the pre-exposure of DC subsets to HBsAg influences the phenotype of NK cells. In the absence of TLR stimulation, NK cells exhibited notable phenotypic changes. However, the significant difference was observed only in the increased frequency of CTLA4^+^ NK cells following coculture with cDC1s preincubated with HBsAg compared with coculture without HBsAg (Figure 4*B*; Supplementary Table 4).

Following TLR stimulation of DC subsets, several alterations in the NK cell phenotype were observed (Figure 4*B*). Interestingly, the pre-exposure of cDC1s to HBsAg significantly increased the frequency of OX40^+^ NK cells, whereas the pre-exposure of pDCs to HBsAg led to significantly decreased frequency of NKG2A^+^ NK cells in the condition of TLR (CpGA) stimulation. Even though this was not significant, we observed tendencies toward decreased TIM3 and NKG2D expression levels as well as increased TIGIT and GITR levels.

When NK cells were cocultured with PanDCs, an increased frequency of TIGIT^+^ NK cells was observed following HBsAg pre-exposed PanDC and Poly(I:C) stimulation (Figure 4*C*). This effect on TIGIT expression was consistent even when the analysis was conducted separately for CD56^dim^CD16^+^ and CD56^bright^CD16^−^ NK cell subsets (Figure 4*C*).

Interestingly, further phenotypic analysis of NK subsets cocultured with different DC subsets revealed distinct responses. The major circulating subset, CD56^dim^CD16^+^ NK cells, exhibited a similar expression profile to the whole NK cell population (Figure 5*A–D*). Notably, we observed that pre-exposure of pDCs to HBsAg significantly reduced the frequency of TIM-3^+^ NK cells and NKG2A^+^ NK cells in the condition of TLR (CpGA) stimulation (Figure 5*D*).

**Figure 5:**
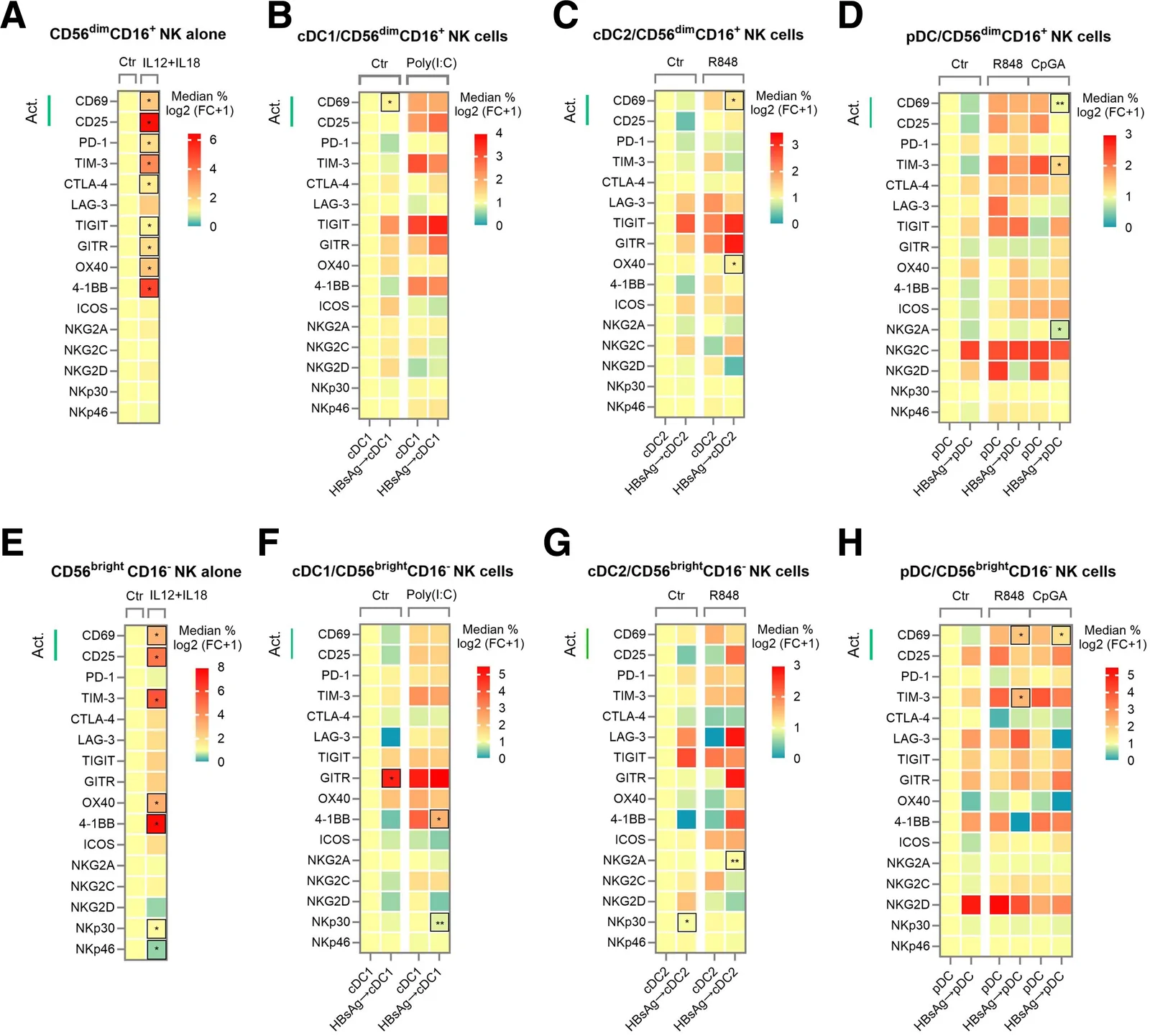
**Phenotyping and activation markers of NK cell subsets.** DCs were exposed or not to HBsAg and further cocultured with NK cells. NK cells’ phenotype was analyzed by flow cytometry. (*A*) Heatmap of median frequencies of CD56^dim^CD16^+^ NK cell subsets in conditions when NK cells were cultured alone, either nonstimulated or stimulated with IL12 and IL18, analyzed by flow cytometry. (*B*) Heatmap of median frequencies of CD56^dim^CD16^+^ NK–positive cells when NK cells were cocultured with HBsAg–pre-exposed cDC1s or not. (*C*) Heatmap of median frequencies of CD56^dim^CD16^+^ NK–positive cells when NK cells were cocultured with HBsAg–pre-exposed cDC2s or not. (*D*) Heat map of median frequencies of CD56^dim^CD16^+^ NK–positive cells when NK cells were cocultured with HBsAg–pre-exposed pDCs or not. (E) Heatmap of median frequencies of CD56^bright^CD16^-^ NK cell subsets in conditions when NK cells were cultured alone, either nonstimulated or stimulated with IL12 and IL18, analyzed by flow cytometry. (*F*) Heatmap of median frequencies of CD56^bright^CD16^-^ NK–positive cells when NK cells were cocultured with HBsAg–pre-exposed cDC1 or not. (G) Heat map of median frequencies of CD56^bright^CD16^-^ NK–positive cells when NK cells were cocultured with HBsAg–pre-exposed cDC2 or not. (*H*) Heatmap of median frequencies of CD56^bright^CD16^-^ NK–positive cells when NK cells were cocultured with HBsAg–pre-exposed pDC or not. Data represents 7 independent experiments with 7 different HDs used for cell isolation. ∗*P* < .05, ∗∗*P* < .01. FC, fold change.

The minor subset, CD56^bright^CD16^−^ NK cells, displayed a different profile when cocultured with DCs preincubated with HBsAg (Figure 5*E–H*). Pre-exposure of cDC1s to HBsAg significantly decreased the frequency of 4-1BB^+^ and NKp30^+^ CD56^bright^CD16^−^ NK cell subsets in condition of Poly(I:C) stimulation (Figure 5*F*), whereas pre-exposure of pDCs to HBsAg led to significantly decreased frequency of TIM-3^+^ NK cells in the condition of R484 stimulation (Figure 5*H*).

To conclude, NK cells cocultured with HBsAg–pre-exposed DCs displayed phenotypic alterations mainly upon stimulation of DCs with TLR-L.

### Hepatitis B Surface Antigen Effects Cytokine and Chemokine Secretion by the Dendritic Cells and Natural Killer Cells

We then studied the impact of HBsAg pre-exposure of DC subsets on the cytokine, chemokine, and soluble ICM profiles of DC and NK cells. The cells were cultured at a 1:10 DC:NK ratio, leading us to attribute the primary contributions in the secretome to NK cells. We analyzed 20 distinct analytes commonly associated with NK cell function. NK cells stimulated with IL12 and IL18 served as a positive control (Figure 6*A*). Heatmaps were generated based on the median fold change of analyte levels relative to control conditions. Our data revealed that preincubation of DC subsets with HBsAg modulates the NK cell secretome, both directly and in response to TLR activation (Figure 6*B*; Supplementary Table 5). Specifically, HBsAg induced the production of proinflammatory chemokines, with a notable increase in CCL3 and CCL4 in cDC1 and NK and cDC2 and NK cocultures (Figure 6*B*). However, under TLR stimulation, HBsAg significantly suppressed CXCL10 (IP-10) production. This reduction was observed in cDC1 and NK cocultures following Poly(I:C) stimulation and in pDC and NK cocultures after R848 stimulation. Notably, only trends were observed in PanDC and NK conditions (Figure 6*C*).

**Figure 6:**
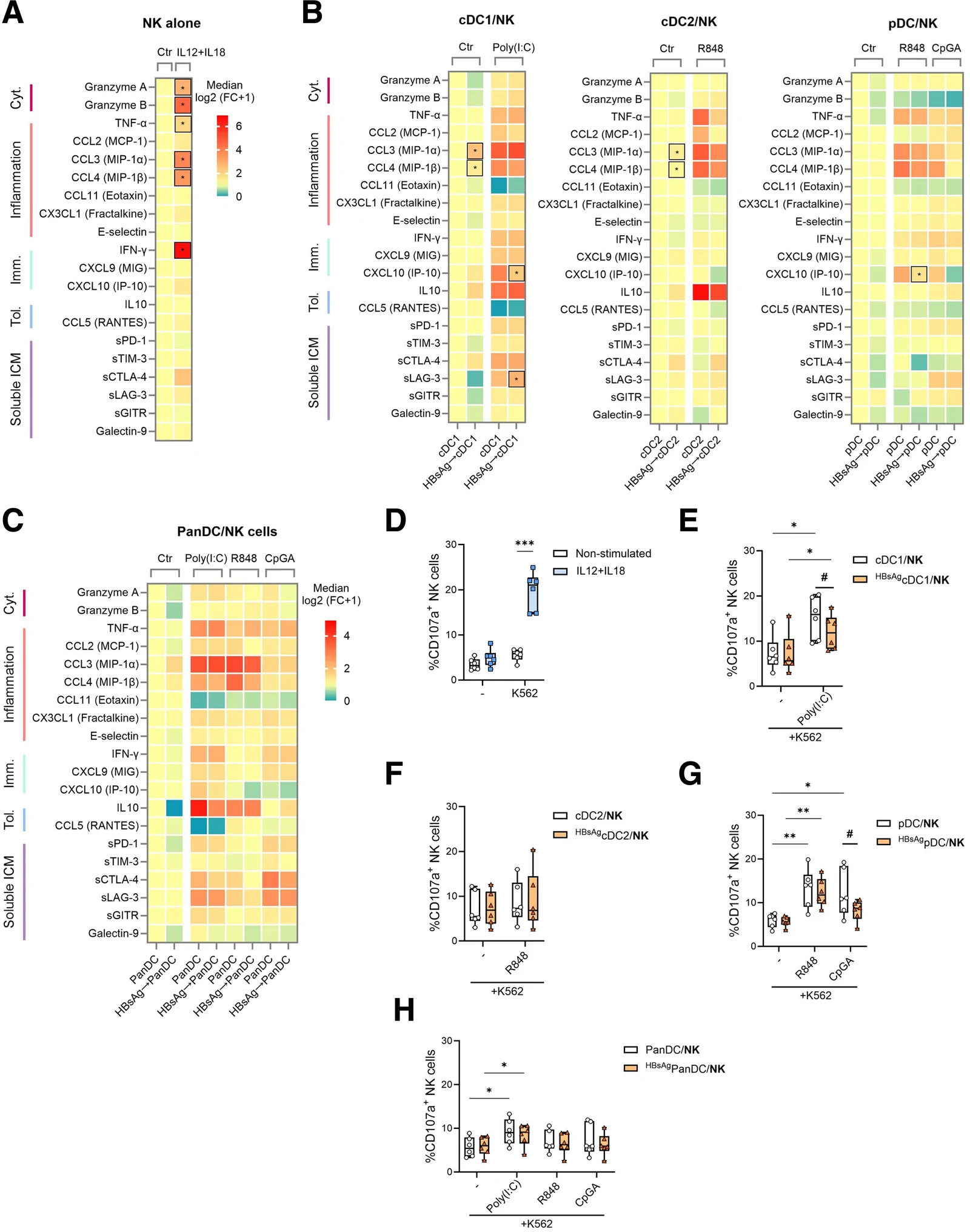
**HBsAg modulates the DC–NK cell secretome and impairs the capacity of DCs to trigger NK cell cytotoxicity.** PanDCs purified from HDs were exposed or not to HBsAg and further cocultured with NK purified from HDs. NK cells’ secretome was further analyzed by Luminex. (*A*) Heatmaps based on the fold change (FC) of the median concentration of cytokines, chemokines, and soluble ICM analyzed in the NK cell secretome. Reference value, no stimulation, was settled as blank. (*B*) Heatmaps based on the FC of the median concentration of cytokines, chemokines, and soluble ICM analyzed in the cDC1–NK, cDC2/NK, and pDC–NK cell secretome. (*C*) Heatmap based on the FC of the median concentration of cytokines, chemokines, and soluble ICM analyzed in the PanDC1–NK cell secretome. Reference value, no stimulation without HBsAg, was settled as blank. *Asterisks* indicate significant differences within subgroups of HBsAg–pre-exposed DCs compared with the condition without HBsAg. ∗*P* < .05. N = 6–7 independent experiments (7 different HDs used for isolation of cells). (*D*) The frequency of CD107a^+^ NK cells cultured alone, stimulated or nonstimulated in the presence (K562) or in the absence (−) of the target cells K562. (*E*) The frequency of CD107a^+^ NK cells after co-culture with cDC1s, cDC2s (*F*), or pDCs (*G*). (*H*) The frequency of CD107a^+^ NK cells after coculture with PanDCs. Data represents 6 independent experiments (6 different HDs used for isolation of cells). *P* value calculated between HBsAg and control condition for each TLR-L condition. ∗*P* < .05, ∗∗*P* < .01, ∗∗∗*P* < .001 were calculated between nonstimulated and stimulated condition, ^#^*P* < .05 was calculated between HBsAg and control condition for each TLR-L condition.

In summary, our results suggest that preincubation of DC subsets with HBsAg reshapes the cytokine and chemokine secretion profiles of NK cells. Notably, this includes a marked reduction in CXCL10 secretion by NK cells following coculture with DC subsets pre-exposed to HBsAg and subsequent TLR stimulation.

### Hepatitis B Surface Antigen Pre-Exposed Dendritic Cell Subsets Have an Altered Capacity to Trigger Natural Killer Cell Cytotoxicity

We next explored the impact of HBsAg on DC-triggered NK cytotoxicity. NK cells were cocultured with DCs, then incubated with K562 target cells for 3 hours, and the surface expression of CD107a, a marker of degranulation, was assessed. Under control conditions, the frequency of degranulating NK cells in the presence of K562 was significantly enhanced by IL12 and IL18 stimulation compared with nonstimulated cells or NK cells cultured without K562 (Figure 6*D*). NK cells cocultured with TLR-stimulated cDC1s or pDCs showed increased CD107a expression compared with cocultures with nonstimulated DCs, whereas TLR-stimulated cDC2s failed to induce NK cell degranulation (Figure 6*E–G*). Notably, only Poly(I:C)-stimulated PanDCs promoted NK cell degranulation (Figure 6*H*).

Crucially, the preincubation of cDC1s with HBsAg partially impaired their ability to stimulate NK cell cytotoxicity (Figure 6*E*). Furthermore, NK cells cocultured with CpGA-stimulated pDCs pre-exposed to HBsAg did not upregulate CD107a expression (Figure 6*G*), aligning with prior observations that CpGA-stimulated pDCs preincubated with HBsAg fail to activate NK cells. In conclusion, pre-exposure to HBsAg impaired the ability of both TLR-3–stimulated cDC1s and TLR-9–stimulated pDCs to promote NK cell cytotoxicity.

### Circulating Hepatitis B Surface Antigen Impairs Plasmacytoid Dendritic Cell Capacity to Stimulate Natural Killer Cell Cytotoxicity

To investigate the clinical relevance of circulating HBsAg levels on DC-mediated cytotoxicity of NK cells, we exposed various DC subsets to serum from HBV-infected patients with differing concentrations of HBsAg. We then assessed how these pre-exposed DCs influenced NK cell phenotype and function.

Notably, the level of HBsAg in the serum used to pretreat pDCs negatively correlated with CD107a expression on NK cells, indicating reduced cytotoxic potential (Figure 7*A* and *B*). This effect was specific to pDCs, as other DC subsets did not show an altered capacity to activate NK cells. An even stronger negative correlation between serum HBsAg levels and NK cell CD107a expression was observed under CpGA-stimulated conditions, consistent with our earlier in vitro findings (Figure 7*C*). To rule out potential effects of actively replicating HBV, we focused our analysis on a subgroup of patients receiving antiviral therapy, characterized by minimal viral load but varying concentrations of HBsAg (Table 1). Again, a negative correlation was observed between HBsAg levels in the serum used to pre-expose pDCs and their ability to promote NK cell cytotoxicity, both in nonstimulated conditions and under TLR9 stimulation (Figure 7*D* and *E*).

**Figure 7:**
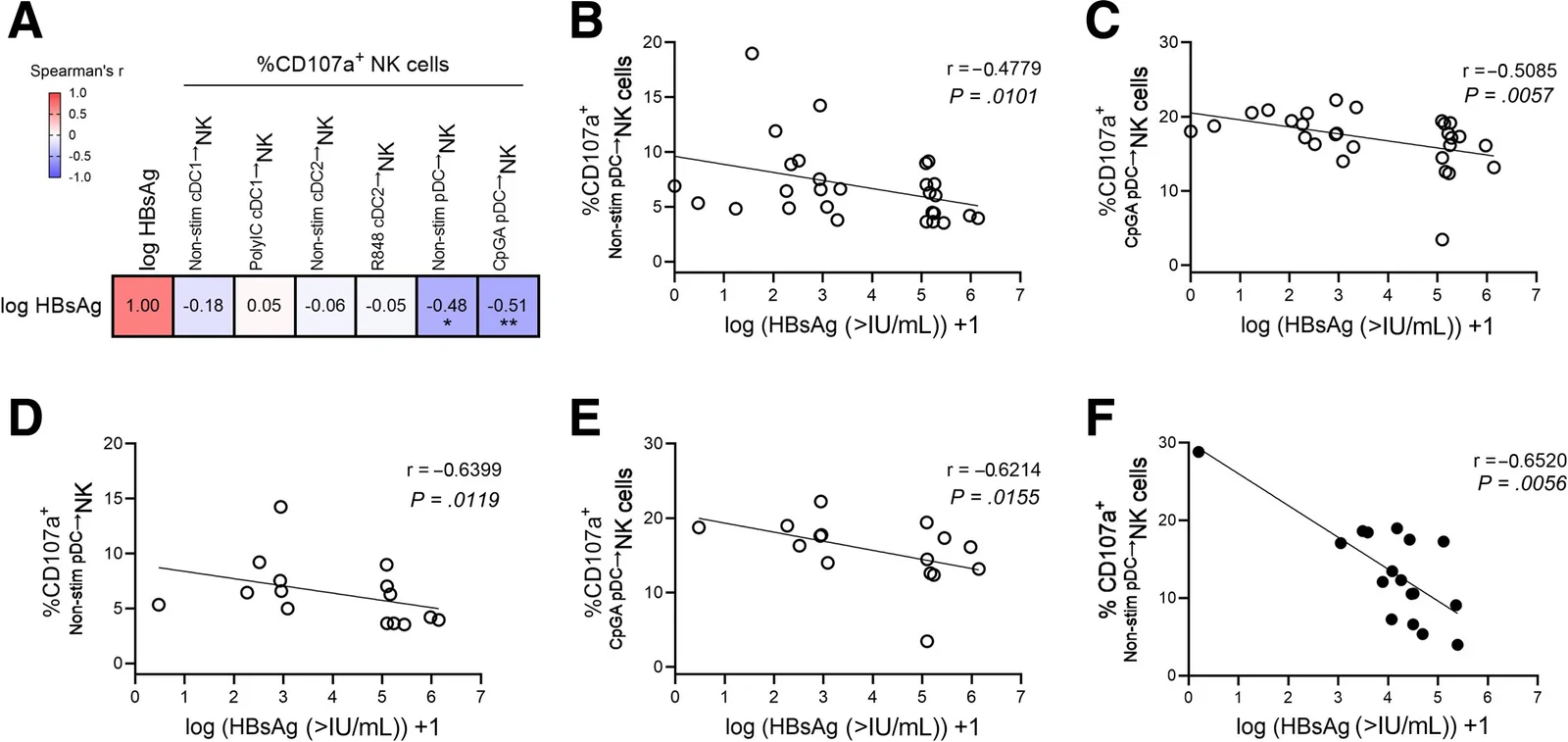
**Circulating HBsAg impairs pDC-mediated NK cell cytotoxicity.** (*A*) DCs purified from HDs were exposed to serum from patients with HBV with different levels of HBsAg and further cocultured with NK purified from HDs. Heatmap is based on the Spearman rank correlation coefficient (r) between the level of HBsAg in the serum used to pretreat DC subsets and CD107a expression on NK cells. HBV-infected patients (N = 28), ∗*P* < .05, ∗∗*P* < .01. (*B*) The correlation between HBsAg level in the serum used to pretreat pDCs with CD107a expression on NK cells, HBV-infected patients (N = 28), and (*C*) under CpGA-stimulated conditions. (*D*) The correlation between HBsAg level in the serum used to pretreat pDCs with CD107a expression on NK cells under CpGA-stimulated conditions, HBV-infected treated patients (N = 15), and (*E*) under CpGA-stimulated conditions. (*F*) pDCs purified from patients with chronic HBV with different levels of HBsAg were cocultured with NK purified from HDs. The correlation was calculated between HBsAg level and CD107a expression on NK cells. Patients with HBV were all treated and HBeAg-negative (N = 17). r, Spearman rank correlation coefficient.

**Table 1:** Characteristics of Patients With HBV

	Cohort for study with serum		Cohort for pDC selection
	Untreated patients with HBV (N = 13)	Treated patients with HBV (N = 15)	Treated patients with HBV (N = 17)
Sex, male/female	7/6	13/2	12/5
Age, *y*	45.0 (21.5–54.0)	43.0 (29.0–68.0)	48.0 (31.0–63.5)
ALT, *U/L*	37.0 (23.5–46.0)	26.0 (24.0–38.0)	30.5 (26.0–35.0)
AST, *U/L*	27.0 (18.5–39.5)	27.0 (24.0–37.0)	26.0 (15.0–43.0)
HBsAg, *IU/mL*	198 (7.4–17,198)	12,304 (86–17,015)	1808 (580–4054)
HBV DNA, *log10 IU/mL*	1.89 (1.13–3.54)	1.0 (1.0–1.04)	1.0 (1.0–1.10)
HBeAg, negative/positive	13/0	15/0	17/0
Antiviral treatment			
Entecavir	0	7	5
Tenofovir	0	8	12

NOTE. Data are presented as number or median (interquartile range).

ALT, alanine transaminase; AST, aspartate transaminase; HBeAg, hepatitis B e antigen; HBsAg, hepatitis B surface antigen; HBV, hepatitis B virus.

Finally, we purified pDCs directly from fresh blood samples of patients with chronic HBV with different serum HBsAg levels (Table 1) and cocultured them with NK cells isolated from healthy donors (HDs). Consistent with our previous observations, we identified a strong negative correlation between serum HBsAg levels and NK cell CD107a expression (Figure 5*F*), revealing the impact of chronic antigen exposure on DC–NK cell interactions.

In conclusion, these findings demonstrate that circulating HBsAg impairs the ability of pDCs to stimulate NK cell cytotoxicity, mainly in a TLR9-dependent manner, highlighting a crucial mechanism of immune dysfunction in chronic HBV infection.

## Discussion

Functional impairments in both DCs^10^, ^11^, ^12^^,^^16^ and NK cells^6^^,^^17^^,^^18^^,^^20^, ^21^, ^22^, ^23^^,^^26^^,^^32^ have been reported in patients with chronic HBV infection. However, the mechanisms underlying these dysfunctions, as well as the potential crosstalk between specific DC subsets and NK cells, remain incompletely understood. Here, we demonstrate that NK cell dysfunction can be mediated by DCs. Although chronic HBV infection is known to alter DC function, the consequences of these alterations for DC interactions with other immune populations are less well-characterized. In this study, we investigated how HBsAg shapes DC subsets, and how these changes influence DC–NK cell interactions and indirectly impact NK cell activation, phenotype, and function.

First, we highlight that HBsAg drastically remodeled the DC secretome, especially cytokines and chemokines involved in the triggering of NK function, as demonstrated both at the RNA and protein levels. HBsAg also alters the capacity of DCs to respond to TLR stimulation. These findings are concordant with what is observed in patients with CHB,^11^^,^^12^^,^^14^^,^^15^ and further suggest that circulating HBsAg levels could directly trigger such modulations.

We then report the effects of HBsAg on the capacity of DCs to activate and regulate NK cells. Our results demonstrate that, under normal conditions, all DC subsets—not treated with HBsAg—can activate NK cells upon TLR stimulation. However, HBsAg disrupts cDC2- and pDC-mediated NK cell activation via the TLR7 and 8 and TLR9 pathways, respectively, as evidenced by reduced expression of the early activation marker CD69. Regarding NK cell cytotoxicity, we observed that cDC1s and pDCs are capable of promoting NK cell degranulation under normal conditions. However, HBsAg preincubation significantly reduced their capacity to trigger NK cell cytotoxicity via TLR3- and TLR9-dependent mechanisms, respectively. The TLR9-dependent inhibitory effect was further validated using serum from HBV-infected patients. Using freshly isolated pDCs from patients with chronic HBV, we observed that pDCs from patients with high serum HBsAg levels displayed a reduced capacity to stimulate NK cell cytotoxicity. These findings further support the detrimental impact of chronic HBsAg exposure on pDC–NK cell interactions and underscore a clinically relevant mechanism by which circulating HBsAg contributes to immune dysregulation in chronic HBV infection.

Overall, our data suggest that the DC–NK crosstalk is highly sensitive to HBsAg. These findings align with previous reports linking high circulating HBsAg levels to impaired immune responses.^19^^,^^33^ Moreover, emerging evidence suggests that HBsAg inhibits DC function following TLR stimulation via CLR- and glycosylation-dependent pathways.^15^ This underscores the importance of further investigating the involvement of TLR and CLR pathways in the disrupted DC–NK crosstalk and the role of HBsAg in immune subversion. TLR7 and TLR8 agonists are currently under active clinical investigation in patients with chronic HBV infection, reflecting their potential to modulate antiviral innate immune responses. However, circulating HBsAg might decrease their efficacy, and the combination with anti-HBsAg antibody might be relevant therapeutic strategy. Notably, in a recent phase II trial, treatment with xalnesiran (a small interfering RNA agent) in combination with the TLR7 agonist ruzotolimod was associated with substantial HBsAg loss, but only in participants with baseline HBsAg levels below 1000 IU/mL.^34^ In conclusion, these efforts underscore the clinical relevance of targeting innate immune sensing pathways to enhance immune control of HBV, while suggesting that baseline HBsAg levels may be an important determinant of therapeutic responsiveness. Our findings provide additional mechanistic insight into how TLR stimulation may shape DC–NK cell crosstalk in the context of HBsAg exposure.

Activation and cytotoxicity of NK cells in patients with CHB has been shown to negatively correlate with HBsAg levels.^19^^,^^23^^,^^26^ Moreover, a recent study suggested that, in patients with HBV and low HBsAg levels, enhanced NK cell activation may contribute to viral clearance.^23^ Our present work further suggest that such HBsAg-dependent defects could be indirect, mediated by HBsAg-subverted DCs.

An important remaining question is whether the HBsAg-mediated alterations in DC function and DC–NK cell crosstalk are reversible following HBsAg reduction or neutralization. Further studies, including analyses of patients receiving HBsAg-lowering therapies, will be necessary to determine whether these immune alterations can be reversed.

One limitation of this study is that it relies on the purity of the cDC1 population, which should more accurately be considered as cDC1-enriched cDCs due to exclusive contamination with the cDC2 population. Nevertheless, our results consistently demonstrate distinct functional effects between cDC1-enriched and highly purified cDC2 populations, which suggests that the observed differences retain biological specificity.

In conclusion, this work provides a detailed view and new insights into the mechanisms underlying DC–NK crosstalk and its disruption in HBV infection. It highlights HBsAg as a critical factor in the dysfunctional pDC–NK interaction, particularly through the impairment of TLR9-mediated responses. Furthermore, HBsAg also disrupts cDC2-mediated NK cell activation via TLR7- and 8-dependent pathways and cDC1-mediated NK cell degranulation via TLR3-dependent pathways.

These findings deepen our understanding of immune dysregulation in chronic HBV infection, highlighting the critical role of all DC subsets in orchestrating effective crosstalk with NK cells. By uncovering the mechanisms underlying DC–NK cell interactions, this work underscores the therapeutic potential of targeting DCs to restore immune competence. Reversing DC dysfunction may represent a promising strategy to reinvigorate NK cell activity and re-establish immune control over HBV.

## Materials and Methods

### Patients With Hepatitis B Virus and Healthy Donors

This study protocol received approval from the Research Ethics Committee of Grenoble University Hospital (CHU-Grenoble) and the “Comité de Protection des Personnes” (CPP) (ID-RCB: 2018-A02164-51). Additionally, it complied with the Institutional Review Board of the French Blood Service (EFS-AuRA, collection AC-2020-3959). All research was conducted in accordance with both the Declarations of Helsinki and Istanbul. Written consent was given in writing by all subjects. Blood samples, including dry tubes for serum and heparinized tubes for cells, were collected from HDs (N = 47) and HBV-infected patients (N = 45). Serum samples were isolated through centrifugation, whereas peripheral blood mononuclear cells (PBMCs) were extracted using Ficoll density gradient centrifugation and preserved at −150 °C until analysis. The clinical characteristics of the patients with HBV are detailed in Table 1.

### Dendritic Cell and Natural Killer Cell Preparation

PanDCs, comprising a mixture of pDCs, cDC1s, and cDC2s, were enriched from thawed PBMCs from HDs by negative selection using the EasySep Human Pan-DC Pre-Enrichment Kit (StemCell) following the manufacturer’s protocol. A fraction of thawed PBMCs was used to separate pDCs via negative selection using the EasySep Human Plasmacytoid DC Isolation Kit (StemCell). cDC subsets (cDC1s and cDC2s) were further purified from PanDCs by positive selection using the CD141 (BDCA-3) MicroBead Kit and the CD1c (BDCA-1) Dendritic Cell Isolation Kit (Miltenyi Biotec), respectively. NK cells were isolated from PBMCs by negative selection using the EasySep Human NK Cell Isolation Kit (StemCell). The purity of DC subsets and NK cells was determined using a multiparametric flow cytometry approach (Figure 8*A* and *B*). Cells were gated based on side scatter and forward scatter, followed by exclusion of doublets and dead cells, and based on CD45 expression (Figure 8*A*). PanDCs, representing all DC subsets, were identified as Lin- HLA-DR^+^ cells. The 3 different DC subsets were then discriminated based on their differential expression of surface markers (Figure 8*A*). cDC1s were further defined as CD11c^dim^ BDCA3^+^, cDC2s as CD11c^bright^ BDCA1^+^, and pDCs as CD11c^−^ BDCA2^+^. The mean purity obtained for PanDCs was 74.11% (range, 41%–88.34%), 93.4% for pDCs (range, 87.4%–99.2%), 35.9% for cDC1s (range, 17.2%–54.4%), and 97.4% for cDC2s (range, 94.0%–99.9%). Therefore, although pDCs and cDC2s can be considered as pure DC subsets, the cDC1 population should rather be regarded as cDC1-enriched cDCs than as a pure cDC1 subset. NK cells were gated on the CD45^+^ population and then identified as CD56^+^ CD3^-^ cells for the determination of purity. The mean purity obtained for NK cells was 96.29% (range, 94.12%–98.99%).

**Figure 8:**
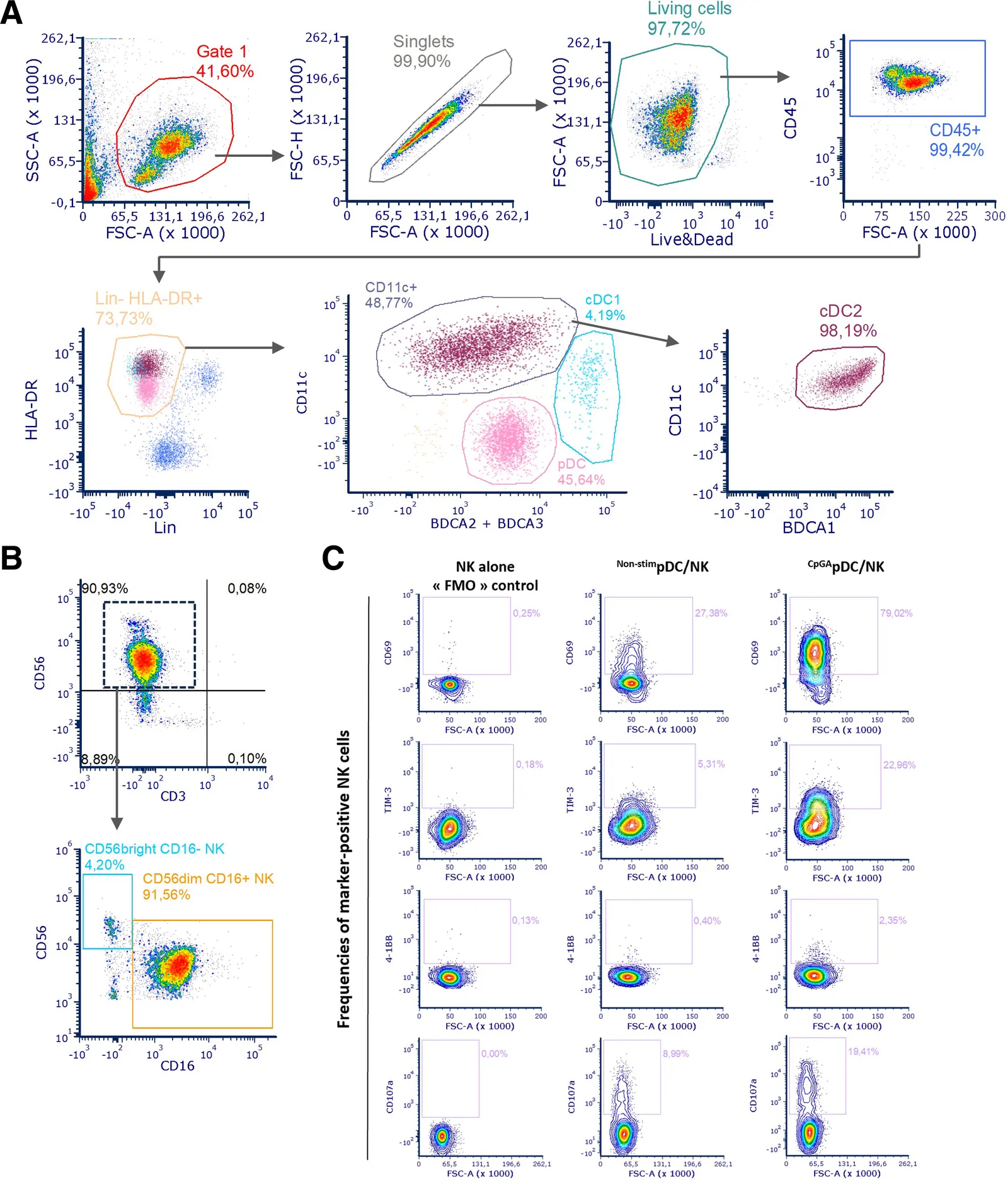
**Flow cytometry gating strategy.** DCs were enriched by negative selection (StemCell) (PanDCs, pDCs) or by positive selection (cDC1s, cDC2s). NK cells were purified by negative selection. (*A*) Using BD FACS DIVA 8 and FCS Express 7 Flow software, debris were first excluded based on size and granularity parameters. Single cells were selected, and dead cells were excluded to select CD45^+^ cells on living cells. PanDCs were identified within CD45^+^ cells as Lin- HLA-DR^+^. Among PanDCs, cDC1s were defined as CD11c^inter^ BDCA3^+^, cDC2s as CD11c^hi^ BDCA1^+^, and pDCs as CD11c- BDCA2^+^. (*B*) For NK cell phenotyping, the gating was carried out in the same way for the DC using FCS Express 7 Flow, and NK cells were then defined as CD56^+^ CD3^-^ cells among CD45^+^ cells. Among NK cells, 2 main subpopulations of NK cells were defined as CD56dim CD16^+^ NK cells and CD56bright CD16- NK cells. (*C*) Expression of CD69, TIM-3, 4-1BB, and CD107a by NK cells from HDs after coculture with nonstimulated (*middle panels*) or stimulated pDCs (*right panels*) measured by flow cytometry (gated on CD56^+^ CD3^-^ NK cells). *Left panels* represent the fluorescence minus one (FMO) control. FSC, forward scatter; SSC, side scatter.

For isolation of pDCs from patients with chronic HBV, PBMCs were isolated directly from fresh blood samples, and pDCs were separated via negative selection using the EasySep Human Plasmacytoid DC Isolation Kit (StemCell).

### Hepatitis B Surface Antigen

Purified HBsAg from human HBsAg-positive serum (MyBioSource, MBS318792) was used at a concentration of 25 μg/mL (eg, 25,000 IU/mL), corresponding to the physiological concentration of HBsAg in serum of patients with HBV.

### RNA Sequencing Analyses of Hepatitis B Surface Antigen–Treated Fluorescence-Activated Cell Sorting–Sorted Pan Dendritic Cells

PanDCs were enriched from HDs’ PBMCs obtained by immunomagnetic negative selection as described above and then stained with anti-CD11c, anti-HLA-DR, and anti-Lin antibodies before undergoing FACS on a BD Aria system to isolate pure PanDC (Lin^−^ HLA-DR^+^) with a purity exceeding 99.8%. Following sorting, PanDCs were cultured for 20 hours in the presence or absence of HBsAg (25 μg/mL) (Figure 1*A*). After incubation, cells were washed with phosphate-buffered saline, and the resulting pellets were resuspended in RNAlater (Sigma-Aldrich) for RNA sequencing analysis. Total RNA was extracted from PanDCs (N = 4/group) using the RNeasy Mini Kit (Qiagen), following the manufacturer’s protocol. RNA quality was assessed using the RNA Integrity Number on an Agilent 2100 Bioanalyzer (Agilent). Messenger RNA libraries were prepared using the SMARTseq2 protocol, as per the manufacturer’s instructions (BGI). A total of 16,367 genes were detected. Data normalization and differential expression analyses were carried out using Dr Tom software.

### Dendritic Cell Culture and Toll-Like Receptor Ligand Stimulation

The culture medium was prepared using RPMI-1640/GlutaMAX (Invitrogen), supplemented with 1% minimal essential medium (100×) nonessential amino acids (Invitrogen), 20 μg/mL gentamycin (Invitrogen), 1 mM sodium pyruvate (Sigma-Aldrich), and 10% human serum. DCs were plated at a density of 2 × 10^6^ cells/mL in the prepared culture medium in 96-well round-bottom plates. Cells were incubated at 37 °C with 5% CO_2_ in the presence or absence of HBsAg (25 μg/mL). After a 4-hour incubation, specific TLR-Ls were used depending on the DC subtype: PanDCs were stimulated or not with the TLR3 agonist Poly(I:C) at 30 μg/mL (Invivogen), the TLR7 and 8 agonist R848 at 1 μg/mL (Invivogen), or the TLR9 agonist CpGA at 1 μM (Invivogen); cDC1s were stimulated with Poly(I:C) or left unstimulated; cDC2s were stimulated with R848 or left unstimulated; pDCs were stimulated with R848, CpGA, or left unstimulated. Cells were incubated for an additional 16 hours (Figure 2*A*).

### Coculture of Dendritic Cells and Natural Killer Cells

DCs—including PanDCs, cDC1s, cDC2s, and pDCs—were purified and cultured in 96-well round-bottom plates in complete RPMI medium as described before. DCs were either preincubated with HBsAg or left untreated for 20 hours at 37 °C in a 5% CO_2_ incubator. For serum incubation experiments, DCs were pretreated for 20 hours with serum samples (50%) obtained from HDs or from treated and untreated patients with HBV exhibiting either low or high levels of circulating HBsAg, under the same incubation conditions. Afterwards, the DCs were washed with RPMI. For the coculture, isolated NK cells were added to the DCs at a DC:NK ratio of 1:10, a proportion commonly used in in vitro studies to model physiological DC–NK interactions. Alternatively, NK cells were cultured alone, either with or without stimulation, using IL12 and IL18. Specific TLR-Ls were used depending on the DC subtype (Figure 3*A*). Following 18 hours of coincubation, supernatants were collected, and cells were split into 2 separate 96-well plates for subsequent assays. One plate was used for NK cell degranulation assays, whereas the other was used for NK cell phenotyping by immunostaining. Washing steps were performed using phosphate-buffered saline containing 2% fetal bovine serum.

### CD107a Degranulation Assay

The leukemia cell line K562, cultured with RPMI 1640 supplemented with 10% of fetal bovine serum and 1% penicillin–streptomycin, was used for cytotoxicity assays. After coculture with DCs, NK cells were incubated with or without K562 target cells with an effector-to-target ratio of 5:1. Concurrently, anti CD107a-FITC antibodies (BD Biosciences) were added. After incubation for 1 hour at 37 °C with 5% CO_2_, BD GolgiStop containing monensin (BD Biosciences) was then added at 0.67 μL/mL, and cells were incubated at 37 °C with 5% CO_2_ for 2 additional hours. NK cell degranulation was assessed by measuring CD107a expression on NK cells using a flow cytometer.

### Phenotypic Analysis of Dendritic Cells by Flow Cytometry

After culturing with ±HBsAg and ±TLR-L, DC subsets were stained using fluorochrome-conjugated antibodies targeting the following human markers: CD11c-PerCP/Cy5.5, HLA-DR-APC-H7, CD86-AF700, TRAIL-BV421 (BD Biosciences), BDCA1/CD1c-PE/Cy7, Lineage cocktail (CD3/CD14/CD16/CD19/CD20/CD56)-BV510, CD45-BV570 (BioLegend), BDCA2/CD303-APC, BDCA3/CD141-APC (Miltenyi Biotec), CD40-PE, CD80-FITC (Beckman) antibodies. Dead cells were excluded using LIVE/DEAD-PE-TxRed viability dye (ThermoFisher). Following staining, cells were fixed with FACS lysing solution (BD) and further analyzed using a BD LSRII Flow Cytometer with BD FACSDiva 8 software (BD Biosciences) and FCS Express-7 Flow software. Within the live CD45^+^ population, subsets were defined as follows: cDC1s: Lin^–^ HLA-DR^+^ CD11c^int^ BDCA3^high^ cells; cDC2s: Lin^–^ HLA-DR^+^ CD11c^high^ CD1c/BDCA1^+^ cells; and pDCs: Lin^–^ HLA-DR^+^ CD11c^–^ BDCA2^+^ cells (Figure 8*A*).

### Phenotypic Analysis of Natural Killer Cells by Flow Cytometry

To analyze NK cell phenotypes after DC–NK coculture, cells were stained using 2 different panels of the following antihuman antibodies: anti-CD45-APC/Cy7 (BioLegend), anti-CD3-PerCP-Cy5.5 (BioLegend), anti-CD56-BV605 (BioLegend), anti-CD16-AF700 (BioLegend) (common to both panels); anti-CD69-PE (BioLegend), anti-CD25-BV510 (BioLegend), anti-OX-40-FITC (BioLegend), anti-4-1BB-BV421 (BioLegend), anti-PD-1-BV711 (BioLegend), anti-TIM-3-BV785 (BioLegend), anti-CTLA-4-PE/Cy7 (BioLegend), anti-GITR-APC (BioLegend) (panel 1); anti-NKp30-PE (BioLegend), anti-NKp46-BV421 (BD Biosciences), anti-NKG2D-BV510 (BioLegend), anti-NKG2C-BV785 (BD Biosciences), anti-NKG2A-BV711 (BD Biosciences), anti-LAG-3-FITC (BioLegend), anti-ICOS-PE/Cy7 (BioLegend), and anti-TIGIT-APC (BioLegend) (panel 2). Corresponding isotype and fluorescence minus one panels were used as controls. Zombie UV Fixable Viability Kit (Biolegend) and LIVE/DEAD Fixable Red Dead Cells Stain (Thermo Fisher) were used to exclude dead cells. Data were acquired on BD LSRII and FACSymphony flow cytometers, collected with BD FACS Diva 6.3.1, and analyzed with FCS Express 7 Flow software. Representative flow cytometry plots illustrating the gating strategy used to identify positive cells are shown in Figure 8*C*.

### Cytokine and Chemokine Secretion Analysis Using Luminex

Supernatants from PanDC cultures and DC–NK culture samples were collected and quantified using Luminex technology. The panel for the PanDC secretome included CCL2 (MCP-1), CCL3 (MIP-1α), CCL4 (MIP-1β), CXCL2 (MIP-2α), CX3CL1 (Fractalkine), TNF-α, IL6, IL8, IL1β, IL23, CXCL11 (I-TAC), CXCL9 (MIG), CXCL10 (IP-10), CXCL1 (GRO-α), IL12p70, IL15, IL18, IL29, IFN-α, IFN-β, CCL5 (RANTES), CCL22 (MDC), CCL17 (TARC), transforming growth factor β, and IL10. The panel for the DC–NK cell secretome included granzyme A, granzyme B, TNF-α, CCL2 (MCP-1), CCL3 (MIP-1α), CCL4 (MIP-1β), CCL11 (Eotaxin), CX3CL1 (Fractalkine), E-selectin, IFN-γ, CXCL9 (MIG), CXCL10 (IP-10), IL10, CCL5 (RANTES), sPD-1, sTIM-3, sCTLA-4, sLAG-3, sGITR, and galectin-9. Measurements were conducted with the MAGPIX 200 instrument and xPONENT software (Bio-Rad), and data were analyzed using the ProcartaPlex Analysis App.

### Statistical Analysis

Statistical analyses were performed using GraphPad Prism 9 software, using 2-way analysis of variance with Dunnett’s or Šídák’s multiple comparison tests, nonparametric Kruskal–Wallis test, Friedman test with Dunn’s multiple comparisons tests***,*** and Wilcoxon matched-pairs signed rank test. The significance threshold was placed at *P* < .05.

All authors had access to the study data and had reviewed and approved the final manuscript.

## Supplementary Material

Supplementary Material

Extended PDF

## Footnotes

Note: To access the supplementary material accompanying this article, visit the full text version at https://doi.org/10.1016/j.jcmgh.2026.101870.
