# Supplementary material for: Hepatitis B Surface Antigen Directly Alters Dendritic Cell–Mediated Natural Killer Cell Responses
Source: Cell Mol Gastroenterol Hepatol. 2026 Aug 29;20(12):101870. doi: 10.1016/j.jcmgh.2026.101870 (PMC13675923; doi:10.1016/j.jcmgh.2026.101870)
Supplement: Supplementary file 1 [file mmc1.pdf]

SUPPLEMENTARY TABLES

Title: Hepatitis B surface antigen directly alters Dendritic cell-mediated Natural Killer cell responses

Lucile Dumolard[1], Marie-Noelle Hilleret[1,2], Mylene Pezet[1], Thomas Decaens[1,2], Philippe Saas[1,3], Evelyne Jouvin-Marche[1], Patrice N. Marche[1], Caroline Aspord[1,3#], Zuzana Macek Jilkova[1,2#]

Supplementary Table 1.
FACS-sorted PanDC from HDs were exposed or not to HBsAg and submitted to RNA sequencing. Normalized read count (NRC) of selected genes significantly modulated in PanDC pre-exposed to HBsAg compared to non-exposed PanDC. Data are median [IQR, 25th–75th percentile], n=4 independent experiments.

PanDCs	No HBsAg	HBsAg	*P Value*
**CCR2**	341.7 [209.7-510.3]	18.1 [4.7-44.4]	0.0188
**IL18**	59.4 [38.2-76.8]	4.7 [1.2-14.6]	0.0336
**CCL2**	45.3 [22.6-63.6]	5.3 [3.1-11.8]	0.0224
**HAVCR2 (TIM3)**	624.8 [564.9-768.9]	290.8 [244.9-492.2]	0.0223
**LGALS9 (Gal9)**	138.1 [105.4-194.4]	83.6 [69.8-113.3]	0.0453
**CXCL1**	6767 [5924-9461]	21806 [14397-26581]	0.0141
**CXCL8 (IL-8)**	3134 [2424-4193]	13963 [9860-17865]	0.0118
**IL12A**	3.7 [0.7-7.6]	15.4 [7.7-18.5]	0.0256
**CCL22 (MDC)**	5185 [2388-6646]	12519 [10787-14507]	0.0188
**PVR (CD155)**	220.5 [203.3-317.2]	432.0 [329.7-698.5]	0.0462
**IL15**	377.5 [277.7-613.8]	732.4 [564.8-1085]	0.0192
**PD-L1**	307.7 [148.9-374.8]	480.6 [374.1-616.8]	0.0123
**4-1BBL**	14.6 [8.6-20.9]	23.9 [17.6-29.6]	0.0368
**CCL19 (MIP-3B)**	47.7 [14.2-88.2]	959.5 [234.0-3128]	0.1826
**CXCL2**	108.3 [81.3-141.0]	603.6 [221.8-1303]	0.1233
**CD80**	19.3 [8.5-40.3]	97.1 [46.8-131.1]	0.0943
**IL12B**	0.0 [0.0-10.3]	7.5 [0.0-31.6]	0.1964
**CD40**	417.7 [285.6-651.5]	1115 [554.0-1177]	0.0754
**MICB**	46.6 [41.7-47.2]	53.4 [46.8-53.4]	0.4108
**ICOSL**	60.7 [24.7-79.7]	83.9 [39.9-141.2]	0.1480
**CD70**	11.6 [7.9-14.5]	16.5 [9.6-28.3]	0.2500
**CD86**	2365 [1721-2942]	3427 [2455-4836]	0.0500
**MICA**	65.2 [31.7-80.4]	68.3 [51.3-122.9]	0.3035
**CXCL10 (IP-10)**	18.7 [7.4-66.0]	11.3 [6.7-29.6]	0.2432
**LAG3**	1.6 [0.4-1.9]	0.17 [0.0-1.0]	0.3214
**CD30**	50.4 [34.6-140.3]	42.4 [29.4-57.6]	0.3134

Supplementary Table 2.
Mean fluorescence intensity (MFI) of surface markers expressed by PanDC, cDC1, cDC2 and pDC without (-) or with HBsAg, stimulated or not by TLR-L. Data are mean±SE. Data are related to the heatmap from Figure 2B.

	**No HBsAg**		**HBsAg**		
		Adjusted P Value (- vs. PolyIC, - vs. R848, - vs. CpGA)		*Adjusted P Value (- vs. PolyIC, - vs. R848, - vs. CpGA)*	*Adjusted P Value (No HBsAg vs. HBsAg)*
**cDC1**					
**CD40 (MFI)**					
-	50152 ± 4008		86846 ± 7663		*0.0004*
PolyIC	79179 ± 10686	*0.1790*	88346 ± 12958	*0.9996*	*0.6283*
R848	121990 ± 12363	*<0.0001*	116626 ± 9369	*0.1613*	*0.8455*
CpGA	66762 ± 9620	*0.6450*	88335 ± 10508	*0.9996*	*0.0241*
**CD80 (MFI)**					
-	1480 ± 105		2121 ± 278		*0.2367*

PolyIC	6506 ± 992	*<0.0001*	8126 ± 930	*<0.0001*	*0.4541*
R848	7173 ± 666	*<0.0001*	5602 ± 282	*0.0004*	*0.0963*
CpGA	1903 ± 258	*0.9550*	2638 ± 382	*0.9268*	*0.0671*
**CD86 (MFI)**					
-	8088 ± 627		13010 ± 1667		*0.0089*
PolyIC	18535 ± 3730	*0.0480*	20292 ± 3714	*0.2614*	*0.3540*
R848	23158 ± 4065	*0.0016*	21804 ± 2053	*0.1256*	*0.9900*
CpGA	12001 ± 2343	*0.7550*	14434 ± 2292	*0.9838*	*0.0916*
**cDC2**					
**CD40 (MFI)**					
-	14643 ± 1586		36035 ± 4835		*0.0021*
PolyIC	88344 ± 11249	*<0.0001*	78455 ± 9235	*0.0006*	*0.7075*
R848	125401 ± 7802	*<0.0001*	106540 ± 8521	*<0.0001*	*0.0022*
CpGA	22288 ± 4345	*0.8793*	37437 ± 5761	*0.9991*	*0.0373*
**CD80 (MFI)**					
-	1158 ± 35		1681 ± 149		*0.0142*
PolyIC	7485 ± 706	*<0.0001*	5289 ± 466	*<0.0001*	*0.0127*
R848	5145 ± 275	*<0.0001*	3421 ± 174	*0.0026*	*0.0002*
CpGA	1499 ± 129	*0.8899*	1869 ± 206	*0.9790*	*0.1207*
**CD86 (MFI)**					
-	6378 ± 609		8089 ± 818		*0.0014*
PolyIC	12737 ± 2349	*0.0134*	11191 ± 1866	*0.4291*	*0.2903*
R848	12370 ± 1598	*0.0222*	10268 ± 979	*0.7089*	*0.0567*
CpGA	9012 ± 1408	*0.5701*	8686 ± 1023	*0.9912*	*0.9663*
**pDC**					
**CD40 (MFI)**					
-	5195 ± 314		10157 ± 1262		*0.0094*
PolyIC	28021 ± 3487	*0.0005*	16601 ± 1845	*0.6485*	*0.0012*
R848	66859 ± 6802	*<0.0001*	62268 ± 6868	*<0.0001*	*0.3928*
CpGA	12506 ± 2548	*0.5498*	13684 ± 2117	*0.9188*	*0.9471*
**CD80 (MFI)**					
-	1174 ± 88		2856 ± 329		*0.0038*
PolyIC	3193 ± 264	*<0.0001*	2562 ± 172	*0.7951*	*0.0073*
R848	3331 ± 321	*<0.0001*	2650 ± 178	*0.9175*	*0.0137*
CpGA	1796 ± 104	*0.2634*	2518 ± 206	*0.7170*	*0.0404*
**CD86 (MFI)**					
-	5917 ± 851		5825 ± 924		*0.9974*
PolyIC	5392 ± 504	*0.9700*	4826 ± 606	*0.8299*	*0.6670*
R848	7790 ± 1021	*0.3874*	6076 ± 821	*0.9965*	*0.0623*
CpGA	5449 ± 849	*0.9784*	5521 ± 937	*0.9939*	*0.9991*
**PanDC**					
**CD40 (MFI)**					
-	12870 ± 1467		32293 ± 3918		*0.0007*
PolyIC	43093 ± 5786	*0.0027*	37475 ± 5466	*0.9231*	*0.3805*
R848	90351 ± 8679	*<0.0001*	78092 ± 8853	*<0.0001*	*0.0676*
CpGA	21402 ± 4094	*0.7312*	33822 ± 4694	*0.9977*	*0.0582*
**CD80 (MFI)**					
-	1298 ± 43		1894 ± 159		*0.0379*
PolyIC	5118 ± 454	*<0.0001*	4127 ± 298	*<0.0001*	*0.0825*
R848	4378 ± 288	*<0.0001*	3099 ± 142	*0.0041*	*0.0001*
CpGA	1587 ± 130	*0.8328*	2039 ± 163	*0.9744*	*0.0299*
**CD86 (MFI)**					
-	6203 ± 543		8052 ± 760		*0.0031*
PolyIC	8613 ± 1466	*0.3891*	8746 ± 1320	*0.9677*	*0.9974*
R848	10033 ± 1205	*0.0636*	8431 ± 883	*0.9944*	*0.0526*

CpGA	8593 ± 1165	*0.3969*	8626 ± 897	*0.9813*	*>0.9999*

Supplementary Table 3.
Median concentration of cytokines and chemokines secreted by PanDC without (-) or with HBsAg exposure, stimulated or not by TLR-L. Data are median [IQR, 25th–75th percentile]. Data are related to the heatmap from Figure 2C.

	**No HBsAg**		**HBsAg**		
		Adjusted P Value (- vs. PolyIC, - vs. R848, - vs. CpGA)		*Adjusted P Value (- vs. PolyIC, - vs. R848, - vs. CpGA)*	*Adjusted P Value (No HBsAg vs. HBsAg)*
**CCL2 (MCP-1)**					
-	16 [0-21]		59.86 [31-242]		*0.0212*
PolyIC	508 [423-828]	*0.0003*	267 [72-351]	*0.8140*	*0.1010*
R848	375 [192-1181]	*0.0001*	147 [72-351]	*0.9808*	*0.1624*
CpGA	492 [185-749]	*0.0010*	375 [187-672]	*0.1350*	*0.9028*
**CCL3 (MIP-1α)**					
-	16 [9-58]		651 [294-1046]		*0.0073*
PolyIC	5899 [2219-8093]	*<0.0001*	4160 [853-9515]	*<0.0001*	*>0.9999*
R848	2723 [1466-3259]	*0.0208*	3786 [2115-6020]	*0.0057*	*0.3788*
CpGA	40 [16-201]	*0.9997*	777 [551-2228]	*0.9207*	*0.0565*
**CCL4 (MIP-1β)**					
-	145 [45-275]		1555 [432-3827]		*0.0136*
PolyIC	5979 [3373-37207]	*0.0052*	9202 [1175-17460]	*0.5486*	*0.8093*
R848	12901 [7048-28408]	*0.0047*	17536 [10945-40763]	*0.0035*	*0.9852*
CpGA	363 [168-1537]	*0.9995*	3663 [1965-7188]	*0.9751*	*0.0449*
**CXCL2 (MIP-2 α)**					
-	35 [13-42]		77 [46-357]		*0.0524*
PolyIC	71 [34-178]	*0.9088*	263 [213-950]	*0.0306*	*0.4596*
R848	172 [126-291]	*0.4110*	420 [373-1079]	*0.0011*	*0.3123*
CpGA	36 [33-49]	*>0.9999*	123 [70-238]	*0.9630*	*0.0916*
**CX3CL1 (Fractalkine)**					
-	1.8 [0.6-6.1]		6.8 [1.6-17.7]		*0.0880*
PolyIC	16.4 [8.4-66.8]	*0.0164*	15.7 [2.1-66.6]	*0.2088*	*0.9857*
R848	3.3 [1.2-30.0]	*0.6810*	9.0 [4.3-49.8]	*0.6566*	*0.2345*
CpGA	6.5 [2.7-40.6]	*0.3989*	14.7 [7.2-53.7]	*0.3293*	*0.1151*
**TNF-α**					
-	3.1 [0-9.9]		27 [19-49.4]		*0.0246*
PolyIC	5434 [1701-17994]	*0.0017*	3508 [590-6392]	*0.2337*	*0.3132*
R848	6094 [3002-18869]	*0.0003*	11619 [8004-17738]	*<0.0001*	*0.5436*
CpGA	5.6 [1.5-79]	*>0.9999*	44 [29-265]	*>0.9999*	*0.1856*
**IL-6**					
-	2.5 [0-44]		512 [121-1168]		*0.0679*
PolyIC	44038 [16574-46986]	*<0.0001*	17642 [5613-44038]	*<0.0001*	*0.5456*
R848	14292 [10175-27929]	*0.0008*	29017 [15887-44038]	*<0.0001*	*0.1199*
CpGA	32 [4.8-127]	*>0.9999*	531 [310-3098]	*0.9996*	*0.1150*
**IL-8**					
-	860 [438-4635]		12223 [6687-14260]		*0.1659*

PolyIC	5035 [3415-15535]	*0.4666*	7100 [467-14551]	*0.7673*	*0.9285*
R848	8385 [3199-9535]	*0.4934*	12124 [6616-13377]	*0.9903*	*0.0616*
CpGA	653 [441-1635]	*0.9764*	13965 [10084-24568]	*0.7751*	*0.0605*
**IL-1β**					
-	0.7 [0-5.8]		19.1 [8.4-46.6]		*0.0293*
PolyIC	1399 [312-3349]	*<0.0001*	420 [102-870]	*0.5411*	*0.1833*
R848	239 [111-1520]	*0.2943*	2458 [1519-2983]	*<0.0001*	*0.0130*
CpGA	5.4 [1.8-16.3]	*>0.9999*	32 [19-79]	*>0.9999*	*0.0684*
**IL-23**					
-	0 [0-7.1]		0 [0-17.2]		*0.8456*
PolyIC	10278 [4452-30662]	*0.3425*	13126 [2469-64642]	*0.3345*	*0.9549*
R848	402 [0-1183]	*>0.9999*	1216 [380-1897]	*>0.9999*	*0.2667*
CpGA	0 [0-746]	*>0.9999*	0 [0-35]	*>0.9999*	*0.6526*
**CXCL11 (I-TAC)**					
-	4.7 [0-13.3]		17 [10-39]		*0.1465*
PolyIC	231 [137-254]	*0.0004*	113 [43-204]	*0.2122*	*0.0947*
R848	146 [94-332]	*0.0002*	160 [56-195]	*0.0751*	*0.4786*
CpGA	92 [17-370]	*0.0033*	61 [23-168]	*0.2706*	*0.5988*
**CXCL9 (MIG)**					
-	6.2 [2.4-15]		17 [8.3-29]		*0.4621*
PolyIC	574 [254-1045]	*<0.0001*	169 [115-604]	*0.0381*	*0.0686*
R848	209 [103-443]	*0.1133*	113 [74-308]	*0.5329*	*0.0642*
CpGA	153 [67-538	*0.0250*	88 [34-559]	*0.3664*	*0.6975*
**CXCL10 (IP-10)**					
-	17 [6.8-71]		59 [22-104]		*0.4375*
PolyIC	2917 [1730-4502]	*<0.0001*	1392 [687-2896]	*0.0073*	*0.1515*
R848	1306 [863-1999]	*0.0038*	1646 [927-2125]	*0.0104*	*0.9996*
CpGA	935 [455-2937]	*0.0024*	718 [51-2286]	*0.1548*	*0.7676*
**CXCL1 (GRO-a)**					
-	9.1 [2.6-41]		395 [233-1084]		*0.1568*
PolyIC	473 [51-742]	*0.8514*	972 [801-3535]	*0.1193*	*0.4742*
R848	643 [429-913]	*0.5709*	1065 [696-1868]	*0.6235*	*0.6338*
CpGA	4.6 [0-8.5]	*>0.9999*	473 [180-1149]	*0.9055*	*0.0995*
**IL-12p70**					
-	0 [0-1.8]		0 [0-0.9]		*0.9669*
PolyIC	2140 [339-5168]	*0.0002*	1086 [112-3142]	*0.0289*	*0.8917*
R848	207 [58-1976]	*0.5856*	1164 [210-4445]	*0.0095*	*0.4709*
CpGA	0 [0-1.9]	*>0.9999*	0 [0-14]	*>0.9999*	*0.6072*
**IL-15**					
-	13 [5.3-33]		30 [12-38]		*0.0377*
PolyIC	19 [16-33]	*0.7842*	19 [12-29]	*0.5566*	*0.9661*
R848	17 [15-31]	*0.7052*	23 [16-37]	*0.9997*	*0.8472*
CpGA	7.7 [4.2-12]	*0.4058*	13 [6.2-28]	*0.2626*	*0.2474*
**IL-18**					
-	0 [0-10]		11 [0-49]		*0.7760*
PolyIC	21 [0-27]	*>0.9999*	23 [11-72]	*0.6050*	*0.7401*
R848	29 [18-43]	*0.9993*	53 [17-143]	*0.6677*	*0.8726*
CpGA	4.3 [0-16]	*>0.9999*	16 [0-27]	*0.4717*	*0.9211*
**IL-29**					
-	0 [0-7.1]		0 [0-17]		*0.8456*
PolyIC	10278 [0-30662]	*0.3425*	13126 [2469-64642]	*0.3425*	*0.9549*
R848	402 [0-1183]	*>0.9999*	1216 [380-1897]	*>0.9999*	*0.2667*
CpGA	0 [0-746]	*>0.9999*	0 [0-35]	*>0.9999*	*0.6526*

**IFN-α**					
-	0 [0-1.9]		3.3 [1-6.4]		*0.1082*
PolyIC	221 [168-549]	*0.0937*	83 [35-243]	*0.8490*	*0.2703*
R848	417 [254-585]	*0.0159*	1034 [540-1887]	*<0.0001*	*0.0567*
CpGA	131 [23-303]	*0.7129*	28 [4.6-269]	*0.8387*	*0.9053*
**IFN-β**					
-	0 [0-2.9]		0 [0-2.9]		*>0.9999*
PolyIC	298 [112-1040]	*0.0031*	553 [170-1171]	*0.0002*	*0.9098*
R848	138 [108-218]	*0.6740*	142 [4.8-455]	*0.3958*	*0.8192*
CpGA	63 [0-223]	*0.8849*	17 [0-102]	*0.9943*	*0.2589*
**CCL5 (RANTES)**					
-	1.1 [0-66]		267 [11-1073]		*0.0670*
PolyIC	43 [0-365]	*0.9733*	30 [0-120]	*0.0863*	*0.9839*
R848	60 [0-221]	*0.9854*	719 [225-1229]	*0.3924*	*0.0173*
CpGA	77 [0-278]	*0.9938*	179 [94-590]	*0.6902*	*0.4045*
**CCL22 (MDC)**					
-	2849 [1843-6888]		6924 [3397-30441]		*0.0650*
PolyIC	3913 [3198-29203]	*0.7442*	4656 [1776-28062]	*>0.9999*	*0.9105*
R848	7492 [2432-17139]	*0.5740*	13396 [4391-21102]	*0.8753*	*0.9984*
CpGA	630 [87-1937]	*0.9356*	3521 [1404-12473]	*0.4134*	*0.1435*
**CCL17 (TARC)**					
-	0.1 [0-6.7]		11 [4.6-16]		*0.0268*
PolyIC	10 [0-18]	*0.0072*	7.7 [10-6.7]	*0.6323*	*0.5162*
R848	6.3 [0-11]	*0.3554*	6.8 [1.4-8.0]	*0.1328*	*0.6032*
CpGA	0 [0-4.0]	*0.9388*	4.1 [1.3-11]	*0.2660*	*0.0983*
**TGF-β**					
-	857 [573-1506]		1114 [664-1500]		*0.9931*
PolyIC	203 [47-1822]	*0.9072*	460 [183-1398]	*0.7672*	*0.9842*
R848	954 [229-2145]	*0.9951*	1154 [374-2090]	*0.9948*	*0.9967*
CpGA	835 [235-1637]	*0.9887*	891 [315-1461]	*0.9273*	*0.9891*
**IL-10**					
-	0.2 [0-3.7]		19.1 [4.5-69]		*0.0425*
PolyIC	796 [66-1276]	*0.1654*	193 [104-632]	*0.8078*	*0.2342*
R848	530 [346-2193]	*0.0009*	2002 [714-3859]	*<0.0001*	*0.0557*
CpGA	4.5 [0-6.7]	*>0.9999*	20 [12-85]	*>0.9999*	*0.0949*

Supplementary Table 4.
Median median frequencies of positive NK cell co-cultured with HBsAg-pre-exposed cDC1, cDC2 or pDC, stimulated or not by TLR-L. Data represents 7 independent experiments, median [IQR, 25th–75th percentile]. Data are related to the heatmap from Figure 4B.

	No HBsAg		HBsAg		
		Adjusted P Value (- vs. PolyIC, - vs. R848, - vs. CpGA)		*Adjusted P Value (- vs. PolyIC, - vs. R848, - vs. CpGA)*	*Adjusted P Value (No HBsAg vs. HB sAg)*
**cDC1/NK cells**					
**PD-1**					
-	0.66 [0.12-0.84]		0.76 [0.23-0.96]		*0.2112*
PolyIC	0.78 [0.26-1.30]	*0.8562*	0.77 [0.25-1.32]	*0.8097*	*0.4284*
**TIM-3**					
-	0.88 [0.58-3.59]		0.58 [0.49-5.55]		*0.7160*
PolyIC	5.41 [2.15-13.0]	*0.0573*	2.58 [1.56-6.46]	*0.4135*	*0.0637*
**CTLA-4**					
-	1.83 [1.20-2.42]		2.53 [1.43-3.17]		*0.0419*
PolyIC	1.63 [1.32-2.57]	*0.4434*	2.16 [2.02-3.61]	*0.8117*	*0.1141*
**LAG-3**					
-	1.47 [1.03-2.02]		1.29 [0.86-1.84]		*0.2654*
PolyIC	1.19 [0.53-1.56]	*0.8267*	1.08 [0.52-1.66]	*0.7884*	*0.2682*
**TIGIT**					
-	3.02 [1.29-80.2]		8.03 [3.88-99.6]		*0.0980*
PolyIC	15.9 [6.11-95.0]	*0.7179*	20.1 [9.77-43.7]	*0.7969*	*0.5528*
**GITR**					
-	1.17 [1.50-1.08]		1.93 [1.57-5.37]		*0.1705*
PolyIC	2.76 [3.68-1.03]	*0.2449*	6.69 [1.39-13.9]	*0.1641*	*0.3398*
**OX40**					
-	1.7 [1.09-2.55]		2.3 [1.36-4.07]		*0.0806*
PolyIC	1.25 [1.16-3.95]	*0.5356*	1.77 [1.50-5.33]	*0.5900*	*0.0494*
**4-1BB**					
-	0.16 [0.13-0.60]		0.19 [0.12-0.45]		*0.3310*
PolyIC	1.22 [0.60-4.32]	*0.0529*	1.25 [0.53-6.02]	*0.0354*	*0.4134*
**ICOS**					
-	2.38 [2.07-8.59]		3.90 [2.35-7.61]		*0.7133*
PolyIC	1.86 [1.74-3.23]	*0.8397*	1.49 [0.89-7.20]	*0.8899*	*0.0953*
**NKG2A**					
-	23.8 [13.4-32.4]		29.4 [13.0-39.4]		*0.8752*
PolyIC	27.1 [19.8-46.7]	*0.3274*	32.0 [16.9-42.9]	*0.6549*	*0.6296*
**NKG2C**					
-	1.74 [0.56-3.94]		1.44 [0.29-3.74]		*0.2313*
PolyIC	2.05 [0.73-4.21]	*0.9011*	1.20 [0.07-3.96]	*0.9817*	*0.1081*
**NKG2D**					
-	1.22 [0.24-1.96]		1.42 [0.30-1.88]		*0.4210*
PolyIC	1.11 [0.34-1.77]	*0.7801*	0.70 [0.13-1.91]	*0.6212*	*0.2838*
**NKp30**					
-	70.6 [55.5-79.6]		67.3 [47.7-77.8]		*0.1110*
PolyIC	77.1 [54.6-81.3]	*0.9262*	73.8 [54.6-77.6]	*0.6503*	*0.9565*
**NKp46**					
-	54.3 [46.4-65.5]		51.9 [38.5-68.5]		*0.3865*
PolyIC	64.3 [58.8-75.0]	*0.1534*	67.8 [50.3-75.2]	*0.1395*	*0.8474*
**cDC2/NK cells**					
**PD-1**					
-	0.67 [0.28-1.07]		0.61 [0.21-0.79]		*0.3092*
R848	0.62 [0.22-1.04]	*0.4257*	0.63 [0.19-0.86]	*0.9464*	*0.8907*
**TIM-3**					
-	1.38 [0.46-2.04]		1.46 [0.43-3.42]		*0.3337*

R848	2.31 [0.71-5.23]	*0.3741*	0.92 [0.49-2.50]	*0.7929*	*0.3243*
**CTLA-4**					
-	2.20 [1.38-3.63]		1.79 [1.31-2.16]		*0.4138*
R848	1.82 [1.07-2.06]	*0.3984*	2.07 [1.64-2.75]	*0.4210*	*0.0575*
**LAG-3**					
-	1.43 [0.60-2.32]		1.43 [2.58-1.11]		*0.1363*
R848	1.95 [1.07-2.46]	*0.5707*	1.35 [2.31-1.21]	*0.5391*	*0.5847*
**TIGIT**					
-	3.65 [1.97-58.1]		18.8 [4.17-48.0]		*0.5980*
R848	12.7 [3.89-95.1]	*0.5438*	28.6 [5.49-86.5]	*0.5633*	*0.7301*
**GITR**					
-	1.56 [0.77-4.65]		2.65 [0.91-3.21]		*0.4171*
R848	4.02 [1.11-9.09]	*0.6535*	11.3 [1.07-38.1]	*0.1444*	*0.0946*
**OX40**					
-	1.68 [1.17-3.63]		1.82 [1.55-3.48]		*0.7083*
R848	1.64 [1.19-2.93]	*0.7865*	1.69 [1.36-4.39]	*0.6946*	*0.2683*
**4-1BB**					
-	0.21 [0.11-0.42]		0.14 [0.10-0.36]		*0.2981*
R848	0.26 [0.12-0.46]	*0.3507*	0.20 [0.13-0.88]	*0.3573*	*0.2083*
**ICOS**					
-	2.76 [1.97-6.73]		4.17 [2.18-7.61]		*0.3505*
R848	3.68 [1.96-10.1]	*0.6915*	4.67 [2.38-8.62]	*0.8864*	*0.5166*
**NKG2A**					
-	30.2 [13.3-36.6]		27.2 [13.7-34.9]		*0.7658*
R848	31.4 [15.7-40.4]	*0.6821*	26.2 [13.8-44.5]	*0.6910*	*0.8306*
**NKG2C**					
-	2.98 [0.55-3.99]		3.47 [0.56-4.39]		*0.8359*
R848	1.9 [0.48-4.81]	*0.9021*	3.40 [0.39-4.67]	*0.8130*	*0.9190*
**NKG2D**					
-	1.54 [0.17-1.97]		1.37 [0.20-2.65]		*0.9093*
R848	1.57 [0.19-2.64]	*0.6951*	0.62 [0.03-1.59]	*0.8651*	*0.1257*
**NKp30**					
-	76.1 [49.3-80.0]		71.9 [51.6-80.7]		*0.6837*
R848	75.0 [52.6-82.5]	*0.9345*	74.0 [44.8-78.8]	*0.9613*	*0.1875*
**NKp46**					
-	53.2 [47.5-70.3]		52.6 [49.6-64.4]		*0.9639*
R848	60.2 [53.2-65.8]	*0.5550*	52.2 [46.8-68.9]	*0.9729*	*0.6088*
**$^{pDC/}$NK cells**					
**PD-1**					
-	0.89 [0.20-1.55]		0.75 [0.62-3.56]		*0.7324*
R848	1.21 [0.26-2.40]	*0.8606*	1.13 [0.61-4.47]	*0.9131*	*0.8995*
CpGA	0.81 [0.30-1.61]	*0.8845*	0.90 [0.56-2.24]	*0.9914*	*0.9114*
**TIM-3**					
-	1.9 [0.73-3.79]		1.10 [0.93-3.39]		*0.9301*
R848	6.32 [2.41-13.1]	*0.0843*	4.44 [3.32-7.81]	*0.4089*	*0.2506*
CpGA	7.35 [3.95-13.6]	*0.0918*	3.21 [1.77-6.80]	*0.6725*	*0.0538*
**CTLA-4**					
-	1.65 [1.10-2.40]		1.55 [1.54-3.34]		*0.4169*
R848	2.41 [1.43-2.82]	*0.9009*	2.12 [1.31-3.52]	*0.9995*	*0.9188*
CpGA	2.46 [1.39-3.53]	*0.7985*	3.25 [1.57-4.36]	*0.7594*	*0.5102*
**LAG-3**					
-	1.59 [1.09-1.92]		1.61 [0.96-1.72]		*0.2783*
R848	4.29 [2.18-9.02]	*0.6986*	1.88 [1.13-2.97]	*0.9927*	*0.3043*
CpGA	1.25 [1.03-2.45]	*0.2712*	1.13 [0.86-1.98]	*0.9381*	*0.7041*
**TIGIT**					
-	15.2 [3.41-92.3]		23.5 [6.18-98.9]		*0.9965*
R848	44.7 [6.72-98.7]	*0.9630*	58.3 [7.17-99.3]	*0.8778*	*0.9530*

CpGA	10.1 [5.80-98.1]	*0.9846*	38.7 [6.13-99.6]	*0.9444*	*0.2710*
**GITR**					
-	2.81 [1.30-6.69]		3.29 [0.40-21.0]		*0.6067*
R848	3.86 [1.63-9.76]	*0.9204*	3.27 [0.76-31.9]	*0.8974*	*0.4895*
CpGA	3.13 [1.47-26.7]	*0.5797*	5.66 [1.80-47.8]	*0.7124*	*0.8158*
**OX40**					
-	2.07 [1.10-2.47]		1.88 [1.78-3.58]		*0.1338*
R848	2.12 [1.26-3.18]	*0.9687*	2.14 [1.72-4.78]	*0.9748*	*0.4852*
CpGA	1.80 [0.91-2.89]	*0.6495*	2.80 [1.21-3.88]	*0.9983*	*0.9962*
**4-1BB**					
-	0.13 [0.12-0.80]		0.17 [0.10-1.10]		*0.5932*
R848	0.22 [0.14-1.24]	*0.5646*	0.33 [0.10-1.19]	*0.7082*	*0.9998*
CpGA	0.32 [0.24-1.47]	*0.2663*	0.40 [0.14-0.96]	*0.8492*	*0.0993*
**ICOS**					
-	4.70 [3.38-9.67]		3.63 [1.26-11.4]		*0.9997*
R848	5.14 [3.87-12.6]	*0.8709*	7.97 [3.26-14.4]	*0.7676*	*0.9964*
CpGA	10.5 [4.03-14.0]	*0.5419*	9.27 [2.58-14.9]	*0.6230*	*0.9807*
**NKG2A**					
-	27.3 [15.0-38.1]		21.8 [14.6-35.0]		*0.7492*
R848	29.7 [21.0-43.4]	*0.5010*	26.3 [19.7-42.1]	*0.5039*	*0.9689*
CpGA	29.2 [23.4-41.5]	*0.4662*	22.4 [17.4-39.0]	*0.9381*	*0.0082*
**NKG2C**					
-	1.59 [0.39-3.54]		2.69 [0.39-5.43]		*0.7707*
R848	3.71 [0.46-5.73]	*0.8957*	2.91 [0.52-5.56]	*0.6344*	*0.9015*
CpGA	3.89 [0.68-5.81]	*0.7137*	2.95 [0.46-5.60]	*0.9823*	*0.2674*
**NKG2D**					
-	0.75 [0.02-1.48]		0.74 [0.19-2.36]		*>0.9999*
R848	1.74 [0.25-3.76]	*0.8221*	0.86 [0.13-8.03]	*0.8287*	*>0.9999*
CpGA	1.51 [0.21-7.67]	*0.4600*	0.41 [0.25-6.91]	*0.9222*	*0.5894*
**NKp30**					
-	73.3 [55.0-81.7]		72.8 [53.4-80.8]		*0.4588*
R848	76.9 [57.8-82.5]	*0.9626*	74.2 [50.0-82.8]	*0.9982*	*0.1325*
CpGA	73.9 [47.8-81.2]	*0.9923*	73.4 [49.7-81.9]	*0.9993*	*0.8606*
**NKp46**					
-	60.3 [57.2-69.5]		56.9 [47.8-61.7]		*0.4210*
R848	73.9 [71.8-82.0]	*0.1480*	71.3 [54.9-74.1]	*0.3066*	*0.1608*
CpGA	71.0 [51.6-74.4]	*0.9580*	60.3 [51.8-67.5]	*0.9176*	*0.9391*

Supplementary Table 5.
Median concentration of cytokines, chemokines and soluble ICM analysed in the cDC1/NK, cDC2/NK and pDC/NK cell secretome. NK cell were co-cultured with HBsAg-pre-exposed cDC1, cDC2 or pDC, stimulated or not by TLR-L. Data represents 6-7 independent experiments, median [IQR, 25th–75th percentile]. Data are related to the heatmap from Figure 6B.

	No HBsAg		HBsAg		
		Adjusted P Value (- vs. PolyIC, - vs. R848, - vs. CpGA)		*Adjusted P Value (- vs. PolyIC, - vs. R848, - vs. CpGA)*	*Adjusted P Value (No HBsAg vs. HB sAg)*
**cDC1/NK cells**					
**Granzyme A**					
-	67.7 [50.3-79.8]		41.9 [54.2-39.7]		*0.5682*
PolyIC	119 [78.2-234]	*0.0838*	136 [95.6-250]	*0.0226*	*0.3097*
**Granzyme B**					
-	16.3 [11.2-33.7]		12.3 [8.11-33.7]		*0.4542*
PolyIC	27.1 [21.23-104]	*0.1672*	42.3 [21.2-94.9]	*0.0499*	*0.7028*
**TNF-α**					
-	14.6 [6.17-15.6]		14.6 [9.29-27.6]		*0.3118*
PolyIC	72.3 [67.2-234]	*0.0021*	86. [41.4-123]	*0.0340*	*0.2376*
**CCL2 (MCP-1)**					
-	95.3 [40.0-112]		94.0 [73.5-108]		*0.7264*
PolyIC	357 [147-641]	*0.0183*	221 [140-604]	*0.1379*	*0.4001*
**CCL3 (MIP-1α)**					
-	12.3 [11.0-19.8]		54.2 [37.5-123]		*0.0247*
PolyIC	310 [57.2-1195]	*0.0232*	413 [89.9-554]	*0.0495*	*0.9625*
**CCL4 (MIP-1β)**					
-	77.0 [61.4-91.1]		132 [125-294]		*0.0120*
PolyIC	925 [264-1093]	*0.0002*	632 [311-1077]	*0.0032*	*0.5808*
**CCL11 (Eotaxin)**					
-	12.0 [8.70-17.1]		10.2 [6.88-19.2]		*0.7678*
PolyIC	0.31[0.00-4.75]	*0.0022*	4.35 [1.60-5.40]	*0.0071*	*0.0579*
**CX3CL1 (Fractalkine)**					
-	7.43 [5.31-18.7]		8.25 [3.92-24.0]		*0.6083*
PolyIC	16.1 [12.1-33.4]	*0.1138*	18.4 [12.1-37.3]	*0.0535*	*0.0868*
**E-selectin**					
-	17511 [4460-22632]		14405 [2883-34798]		*0.7120*
PolyIC	26619 [9097-46068]	*0.3021*	26258 [11459-48597]	*0.2756*	*0.0804*
**IFN-γ**					
-	55.0 [38.0-83.3]		54.1 [39.2-105]		*0.6507*
PolyIC	240 [163-853]	*0.0247*	266 [1650-353]	*0.1068*	*0.2315*
**CXCL9 (MIG)**					
-	29.5 [23.5-48.1]		29.5 [23.0-51.2]		*0.7775*
PolyIC	113 [219-87.6]	*0.0313*	109 [48.6-178]	*0.0360*	*0.8945*
**CXCL10 (IP-10)**					
-	17.3 [14.5-28.7]		31.0 [21.6-33.1]		*0.0861*
PolyIC	229 [57.7-663]	*0.0065*	65.1 [16.7-292]	*0.2170*	*0.0280*
**IL-10**					
-	0.25 [0.12-0.43]		0.76 [0.51-1.82]		*0.1739*
PolyIC	4.33 [0.50-9.06]	*0.1282*	5.73 [1.68-12.4]	*0.0354*	*0.2926*
**CCL5 (RANTES)**					
-	492 [443-709]		602 [4402-688]		*0.6472*
PolyIC	15.5 [0.00-236]	*0.0114*	68.1 [0.00-274]	*0.0235*	*0.5109*
**sPD-1**					
-	35.0 [32.3-62.8]		35.0 [19.4-65.4]		*0.9598*
PolyIC	103 [88.6-207]	*0.0058*	96.2 [77.7-210]	*0.0023*	*0.1984*
**sTIM-3**					
-	551 [471-816]		429 [353-1010]		*0.9328*
PolyIC	893 [571-1319]	*0.1162*	939 [708-1335]	*0.0549*	*0.3187*
**sCTLA-4**					
-	172 [0.00-1174]		378 [167-1171]		*0.8574*
PolyIC	1175 [203-1532]	*0.1694*	1175 [616-1948]	*0.0881*	*0.3308*
**sLAG-3**					

-	87.8 [54.2-162]		20.7 [0.00-54.2]		*0.1512*
PolyIC	413 [270-468]	*0.0001*	460 [288-596]	*<0.0001*	*0.0465*
**sGITR**					
-	896 [328-960]		518 [340-1236]		*0.8931*
PolyIC	1501 [877-1884]	*0.0169*	1535 [863-2031]	*0.0169*	*0.9204*
**Galectin-9**					
-	91.8 [49.7-120]		72.9 [44.8-98.5]		*0.1911*
PolyIC	128 [85.1-144]	*0.3393*	98.5 [70.4-122]	*0.1317*	*0.4452*
**cDC2/NK cells**					
**Granzyme A**					
-	55.2 [32.5-67.6]		56.1 [41.9-61.4]		*0.4332*
R848	59.9 [45.3-65.7]	*0.7868*	58.5 [48.6-139]	*0.6344*	*0.8525*
**Granzyme B**					
-	18.2 [8.11-40.9]		16.0 [11.2-36.7]		*0.9720*
R848	25.6 [14.0-39.5]	*0.3845*	30.4 [14.0-59.5]	*0.2535*	*0.8040*
**TNF-α**					
-	10.9 [7.63-26.4]		12.0 [5.39-19.2]		*0.3017*
R848	221 [62.2-276]	*0.0035*	36.1 [16.7-294]	*0.0523*	*0.1122*
**CCL2 (MCP-1)**					
-	58.8 [37.7-80.6]		60.1 [42.7-124]		*0.0822*
R848	355 [139-698]	*0.0514*	73.1 [57.7-1222]	*0.1609*	*0.3462*
**CCL3 (MIP-1α)**					
-	13.9 [7.26-22.5]		22.7 [12.4-33.6]		*0.0169*
R848	376 [126-541]	*0.0037*	168 [75.8-627]	*0.0144*	*0.2812*
**CCL4 (MIP-1β)**					
-	72.6 [63.3-125]		95.8 [82.3-163]		*0.0123*
R848	1580 [539-2464]	*0.0035*	771 [178-2823]	*0.0287*	*0.1277*
**CCL11 (Eotaxin)**					
-	11.8 [7.09-19.2]		12.1 [9.21-16.7]		*0.7248*
R848	8.30 [4.75-12.2]	*0.0861*	6.88 [4.48-8.25]	*0.0454*	*0.4490*
**CX3CL1 (Fractalkine)**					
-	8.66 [4.07-19.3		8.91 [6.50-24.0]		*0.4818*
R848	7.84 [3.79-24.60]	*0.8074*	7.96 [6.56-24.8]	*0.9908*	*0.8478*
**E-selectin**					
-	18630 [4064-30831]		16486 [5276-34859]		*0.3949*
R848	15923 [2948-36214]	*0.9807*	16378 [4723-39918]	*0.9705*	*0.6885*
**IFN-γ**					
-	58.8 [37.2-108]		51.1 [43.2-91.6]		*0.8714*
R848	87.6 [51.1-105]	*0.5301*	66.5 [32.3-132]	*0.6964*	*0.6297*
**CXCL9 (MIG)**					
-	26.3 [18.9-58.2]		37.5 [25.8-51.9]		*0.5068*
R848	35.8 [19.4-57.4]	*0.5459*	30.6 [24.6-46.6]	*0.9459*	*0.5504*
**CXCL10 (IP-10)**					
-	11.5 [9.21-23.3]		11.5 [8.05-21.4]		*0.6031*
R848	13.2 [4.54-24.5]	*0.9938*	7.11 [4.94-133]	*0.2309*	*0.1958*
**IL-10**					
-	0.12 [0.00-0.43]		0.25 [0.00-0.50]		*0.3363*
R848	11.6 [0.90-46.8]	*0.0755*	4.43 [0.88-42.1]	*0.0868*	*0.8234*
**CCL5 (RANTES)**					
-	701 [503-1072]		779 [511-905]		*0.8559*
R848	688 [312-1028]	*0.6754*	517 [439-710]	*0.3162*	*0.4639*
**sPD-1**					
-	44.4 [18.8-62.8]		40.0 [32.3-78.5]		*0.6888*
R848	51.6 [37.7-63.7]	*0.8513*	41.4 [24.6-126]	*0.7335*	*0.6329*
**sTIM-3**					
-	624 [377-806]		737 [477-992]		*0.3466*
R848	547 [409-992]	*0.9952*	608 [377-1269]	*0.8662*	*0.4557*
**sCTLA-4**					
-	203 [167-1175]		490 [0.00-861]		*0.8607*
R848	172 [0.00-1737]	*0.7621*	597 [0.00-1174]	*0.4884*	*0.6667*
**sLAG-3**					
-	54.2 [20.7-162]		76.9 [20.7-210]		*0.7184*
R848	76.9 [20.7-125]	*0.6769*	107 [76.9-152]	*0.5754*	*0.4886*

**sGITR**					
-	627 [326-1486]		662 [402-1338]		*0.6434*
R848	764 [486-1262]	*0.9550*	966 [402-1208]	*0.9563*	*0.7908*
**Galectin-9**					
-	89.8 [41.6-168]		122 [47.0-138]		*0.9918*
R848	58.9 [38.7-121]	*0.5136*	98.5 [57.7-104]	*0.5555*	*0.9004*
**pDC/NK cells**					
**Granzyme A**					
-	63.8 [53.6-73.3]		54.7 [53.5-65.7]		*0.1336*
R848	78.0 [61.92 -95.9]	*0.8143*	75.1 [59.9-87.9]	*0.7301*	*0.9309*
CpGA	74.6 [59.9-105]	*0.9562*	71.1 [65.7-109]	*0.7037*	*0.9757*
**Granzyme B**					
-	235 [52.6-693]		158 [37.6-670]		*0.0992*
R848	149 [83.6-721]	*0.8820*	106 [59.8-397]	*0.5097*	*0.2075*
CpGA	60.5 [38.1-184]	*0.1051*	42.5 [21.2-127]	*0.1482*	*0.3060*
**TNF-α**					
-	17.8 [6.95-23.1]		12.4 [6.95-29.7]		*0.7293*
R848	117 [70.3-950]	*0.0143*	105 [36.0-585]	*0.2380*	*0.3980*
CpGA	50.6 [24.2-147]	*0.8646*	59.3 [23.4-70.7]	*0.9857*	*0.6390*
**CCL2 (MCP-1)**					
-	60.9 [51.0-128]		60.5 [36.3-121.7]		*0.1815*
R848	56.0 [54.3-183]	*0.9977*	52.1 [36.3-76.2]	*0.9157*	*0.4461*
CpGA	102 [80.0-349]	*0.2039*	126 [67.5-231]	*0.5429*	*0.8440*
**CCL3 (MIP-1α)**					
-	15.2 [10.2-20.8]		16.3 [11.0-68.4]		*0.3906*
R848	168 [99.2-712]	*0.0084*	115 [49.1-510]	*0.1409*	*0.2650*
CpGA	61.1 [40.5-303]	*0.3237*	27.3 [15.2-81.4]	*0.9868*	*0.2978*
**CCL4 (MIP-1β)**					
-	68.4 [43.5-128]		78.4 [48.3-225]		*0.5573*
R848	1135 [607-3926]	*0.0167*	648 [414-3309]	*0.0925*	*0.4044*
CpGA	591 [216-1679]	*0.2246*	87.8 [77.0-308]	*0.9586*	*0.2369*
**CCL11 (Eotaxin)**					
-	10.5 [8.82-16.0]		10.7 [9.00-19.1]		*0.5080*
R848	7.87 [4.75-13.0]	*0.3477*	6.86 [5.40-16.6]	*0.4152*	*0.4559*
CpGA	7.33 [2.41-17.1]	*0.4537*	6.77 [2.29-18.1]	*0.2685*	*>0.9999*
**CX3CL1 (Fractalkine)**					
-	8.91 [5.64-25.1]		8.74 [5.65-27.1]		*0.5311*
R848	10.2 [7.35-26.1]	*0.9662*	9.72 [6.67-31.6]	*0.9080*	*0.7612*
CpGA	14.6 [12.4-38.6]	*0.4377*	12.1 [9.96-36.9]	*0.5891*	*0.9333*
**E-selectin**					
-	15757 [5153-41059]		15217 [4187-39434]		*0.9623*
R848	17771 [5261-34495]	*0.9935*	20929 [3792-399918]	*0.9909*	*0.5028*
CpGA	25088 [10880-53810]	*0.7265*	19786 [5634-51460]	*0.7173*	*0.9983*
**IFN-γ**					
-	51.1 [31.1-119]		55.0 [26.5-132]		*0.9744*
R848	92.6 [66.4-116]	*0.8635*	123 [31.1-149]	*0.6283*	*0.7576*
CpGA	170 [35.2-196]	*0.1939*	132 [55.0-260]	*0.0535*	*0.6086*
**CXCL9 (MIG)**					
-	33.4 [21.5-63.2]		29.7 [25.9-60.6]		*0.9998*
R848	45.3 [28.7-64.7]	*0.9224*	37.6 [26.8-72.1]	*0.9406*	*0.9954*
CpGA	66.0 [33.6-117]	*0.0588*	46.9 [38.1-99.3]	*0.2633*	*0.5310*
**CXCL10 (IP-10)**					
-	29.3 [16.4-109]		21.5 [9.21-49.3]		*0.4294*
R848	199 [97.0-470]	*0.0872*	63.4 [13.3-190]	*0.7485*	*0.0387*
CpGA	110 [10.3-321]	*0.5855*	11.5 [2.78-38.2]	*0.8939*	*0.1893*
**IL-10**					
-	0.24 [0.00-0.28]		0.24 [0.12-0.28]		*>0.9999*
R848	0.28 [0.12-0.76]	*0.4029*	0.24 [0.12-0.50]	*0.4681*	*0.9967*
CpGA	0.38 [0.50-0.16]	*0.5947*	0.43 [0.00-0.73]	*0.6929*	*0.9909*
**CCL5 (RANTES)**					
-	741 [533-782]		559 [458-822]		*0.9935*
R848	597 [354-893]	*0.9737*	555 [508-585]	*0.8937*	*0.9984*

CpGA	501 [489-833]	*0.9966*	427 [354-601]	*0.4771*	*0.2046*
**sPD-1**					
-	43.6 [21.6-109]		41.7 [25.6-117]		*0.9987*
R848	53.0 [24.3-65.4]	*0.9928*	56.3 [23.0-135]	*0.9160*	*0.5295*
CpGA	84.7 [38.2-159]	*0.4276*	89.9 [41.8-212]	*0.2305*	*0.5811*
**sTIM-3**					
-	686 [353-1219]		564 [401-1194]		*0.8942*
R848	649 [593-938]	*0.9787*	656 [322-1384]	*0.9808*	*0.9718*
CpGA	733 [698-1464]	*0.7696*	694 [521-1511]	*0.7012*	*0.9882*
**sCTLA-4**					
-	854 [0-1171]		539 [167-661]		*0.4087*
R848	861 [34.4-1174]	*0.9729*	236 [34.4-894]	*0.9958*	*0.2179*
CpGA	597 [172-2356]	*0.4037*	661 [236-1532]	*0.2711*	*0.9964*
**sLAG-3**					
-	87.9 [51.4-162]		51.4 [29.6-125]		*0.3143*
R848	76.9 [51.4-210]	*0.9580*	83.5 [54.2-140]	*0.8212*	*0.9694*
CpGA	311 [245-382]	*0.0004*	297 [245-412]	*0.0001*	*0.9774*
**sGITR**					
-	647 [301-1268]		886 [328-1211]		*0.9966*
R848	406 [280-1327]	*0.9896*	807 [322-1338]	*0.9716*	*0.6382*
CpGA	1045 [322-1530]	*0.8829*	1238 [443-1660]	*0.5422*	*0.4678*
**Galectin-9**					
-	96.8 [55.0-104]		73.4 [61.5-118]		*0.9925*
R848	79.0 [52.4-88.5]	*0.7614*	56.0 [47.0-98.5]	*0.4653*	*0.8912*
CpGA	72.0 [51.0-105]	*0.9792*	63.9 [54.3-112]	*>0.9999*	*0.8596*
